# A point-based system to determine authorship eligibility in a large clinical trial: Insights from the ISCHEMIA trial’s authorship nomination system

**DOI:** 10.1017/cts.2025.10214

**Published:** 2025-12-26

**Authors:** Shari Esquenazi-Karonika, Judith S. Hochman, June Lyo, Mark Xavier, Sean M. O’Brien, Stavroula Boumakis, Anna Naumova, Patenne D. Mathews, Jerome L. Fleg, David J. Maron

**Affiliations:** 1 Cardiovascular Clinical Research Center, NYU Grossman School of Medicine, New York City, NY, USA; 2 Autodesk, San Francisco, CA, USA; 3 Duke Clinical Research Institute, Durham, NC, USA; 4 National Heart, Lung and Blood Institute, Bethesda, MD, USA; 5 Stanford Prevention Research Center, Stanford University School of Medicine, Stanford, CA, USA

**Keywords:** Authorship, publishing, multicenter studies

## Abstract

**Background:**

In team science, invitation to writing groups can be subjective and secretive. Confronted with this challenge in the ISCHEMIA trial, with 320 participating sites, 4 coordinating centers, 6 core laboratories, and numerous committees, we adapted an existing model to develop a transparent, objective, and equitable method for determining authorship eligibility.

**Methods:**

We developed a scoring system based on site performance of tasks critical to trial success that meet the ICJME criteria for authorship. Sites were ranked according to points earned, and points required for potential authorship were communicated to all sites. Site investigators were surveyed for their manuscript topic preferences for writing groups. Beyond the point-based system, authorship positions were also reserved for trial contributors who were not site investigators.

**Results:**

To date, 50 original, peer-reviewed ISCHEMIA trial manuscripts have been published. In total, 208 authors from 33 countries participated in at least one publication. Of the 87 sites that randomized at least 15 participants over a mean of 5 years, 72% had authorship positions across published manuscripts. Surveys were sent to 334 site investigators and 27% responded. Among respondents, 61% indicated that ISCHEMIA was the first trial they had worked on with performance-based criteria for authorship invitation. Respondents agreed the system was transparent (81%), objective (83%), and equitable for early career researchers (70%) and underrepresented minorities in research (57%).

**Conclusions:**

ISCHEMIA employed a point-based authorship eligibility system that most authors found objective, merit-based, transparent, and equitable. Implementation of such a system should be considered for team science publications.

## Background

Authorship in peer-reviewed medical journals is an acknowledgment of an individual’s contribution to research. According to the International Committee of Medical Journal Editors (ICMJE), which provides guidance on authorship and non-authorship recognition, manuscript authorship should reflect each of the following criteria: 1) individuals have made substantial contributions to the conception or design of the work or the acquisition, analysis, or interpretation of data for the work; 2) they have drafted or critically revised the work for important intellectual content; 3) they have provided final approval of the version to be published; and 4) they agree to be accountable for all aspects of the work, therefore ensuring that inquiries related to the validity or integrity of any component of the work are appropriately inspected and resolved [1].

Team science is a collaborative effort to address a scientific challenge that leverages the strengths and expertise of professionals, oftentimes trained in different fields [2]. In team science, offering an opportunity for authorship to those who can meet all ICMJE criteria on the masthead can be subjective and secretive, potentially contributing to competition or ill will among collaborators. Authorship determined by the type and level of contribution can be complicated in large team settings, as the work is distributed among many, and it can be difficult to ascertain who completed which aspects of the research [3]. Scientific journals also have varying requirements regarding disclosure of author contributions to manuscripts, adding another layer of subjectivity and secrecy as to whether all authors met authorship criteria and to what degree [4]. Group authorship, which refers to recognizing a team of researchers by their group name on the byline of a manuscript, has been steadily increasing, indicating that large research teams are using this method to acknowledge collaborators [5,6]. This practice has its own challenges, however, as the organizational formats, which vary by journal, can make it challenging to discern who contributed what aspects of the research to the final manuscript.

### ISCHEMIA trial organization

ISCHEMIA was an international comparative effectiveness trial that explored whether patients with chronic coronary disease and moderate or severe ischemia had better clinical outcomes when they received initial invasive management added to guideline-directed medical therapy compared with initial guideline-directed medical therapy alone.

### ISCHEMIA publications committee

The ISCHEMIA Publications Committee was established to facilitate the dissemination of the maximum amount of information from the trial in a scientifically sound and ethically responsible fashion. The Committee had the following responsibilities: 1) setting publication strategy and appropriate timelines; 2) preventing conflict with and/or the duplication of publications and presentations; 3) reviewing and setting priorities for manuscripts and presentations; 4) establishing writing group members for each publication (identifying a chair, appointing a Publications Committee liaison, enlisting the assistance of appropriate additional experts as deemed appropriate and ensuring all authors met ICMJE criteria; 5) monitoring the progress of each manuscript to ensure publication in a timely fashion; 6) reviewing, editing, and approving all manuscript proposals, analysis plans, presentations, and publications prior to submission; 7) reviewing and approving each writing group’s choice of an appropriate journal for publication (and identifying alternative choices, if needed); 8) ensuring that all NHLBI policies pertaining to publications and presentations are followed; and 9) reviewing and approving ISCHEMIA trial press releases in conjunction with the NHLBI and Statistical and Data Coordinating Center (see ISCHEMIA Publications Committee Policy in the Supplementary Appendix). The Publications Committee was composed of members selected from the Executive Committee. A Publications Coordinator was employed to support the administration of publication-related efforts, such as the circulation of manuscript proposals and manuscript drafts, the collation of feedback, general tracking of ISCHEMIA manuscripts, communication with co-authors, and the submission of manuscripts to journals. A Publications Manager was employed to oversee publication operations. Given the scale of the trial and the geographic distribution of international investigators, a centralized email account was created to receive correspondence related to all ISCHEMIA manuscripts and presentations

## Methods

### Ranking process for selection of site investigators for writing groups

ISCHEMIA investigators were nominated for authorship according to a scoring system developed by the trial’s lead biostatistician with input from the Publications Committee. Through this system, each site earned points determined by the following criteria: 1) number of participants randomized, randomization rate, and percent of women randomized (Veterans Administration sites were exempt from the latter criterion), 2) data quality, completion, and submission, and 3) adherence to the protocol (e.g., adherence to assigned strategy, achievement of medical therapy and revascularization goals, and proportion of participants that met all eligibility criteria, including stress tests that were deemed by core laboratory review to have at least moderate ischemia). The criteria are presented in Table 1. In the event that multiple sites received the same score in this system, the site with the higher total number of randomizations was chosen without regard to randomization rate. If still tied, the decision would be settled by a flip of a coin by the Chair of the Publications Committee, though this was never necessary. The number of points required to earn authorship, presented in Table 2, was communicated to all sites at investigator meetings, in newsletters, and in emails to sites. In a parallel process, all site investigators were surveyed to collect their topic preferences for writing groups from a list of topics generated by the Publications Committee.

**Table 1.** Criteria used to determine site score

**Randomization**
Number randomized
Randomization rate
% of women randomized*
**Data**
Quality
Completion
Submission
**Protocol Adherence**
Adherence to assigned strategy
Achievement of medical therapy and revascularization goals
Proportion of participants that meet all eligibility criteria, including stress tests that are deemed by the core labs to meet ischemia eligibility criteria

* US Department of Veteran Affairs sites were exempted.

**Table 2.** Authorship point system

Author	Points	Spots Available
Lead or Senior Author	40	1
Second Author	20	1
Third Author	15	1
Co-Author	10	21

**Figure 1.**
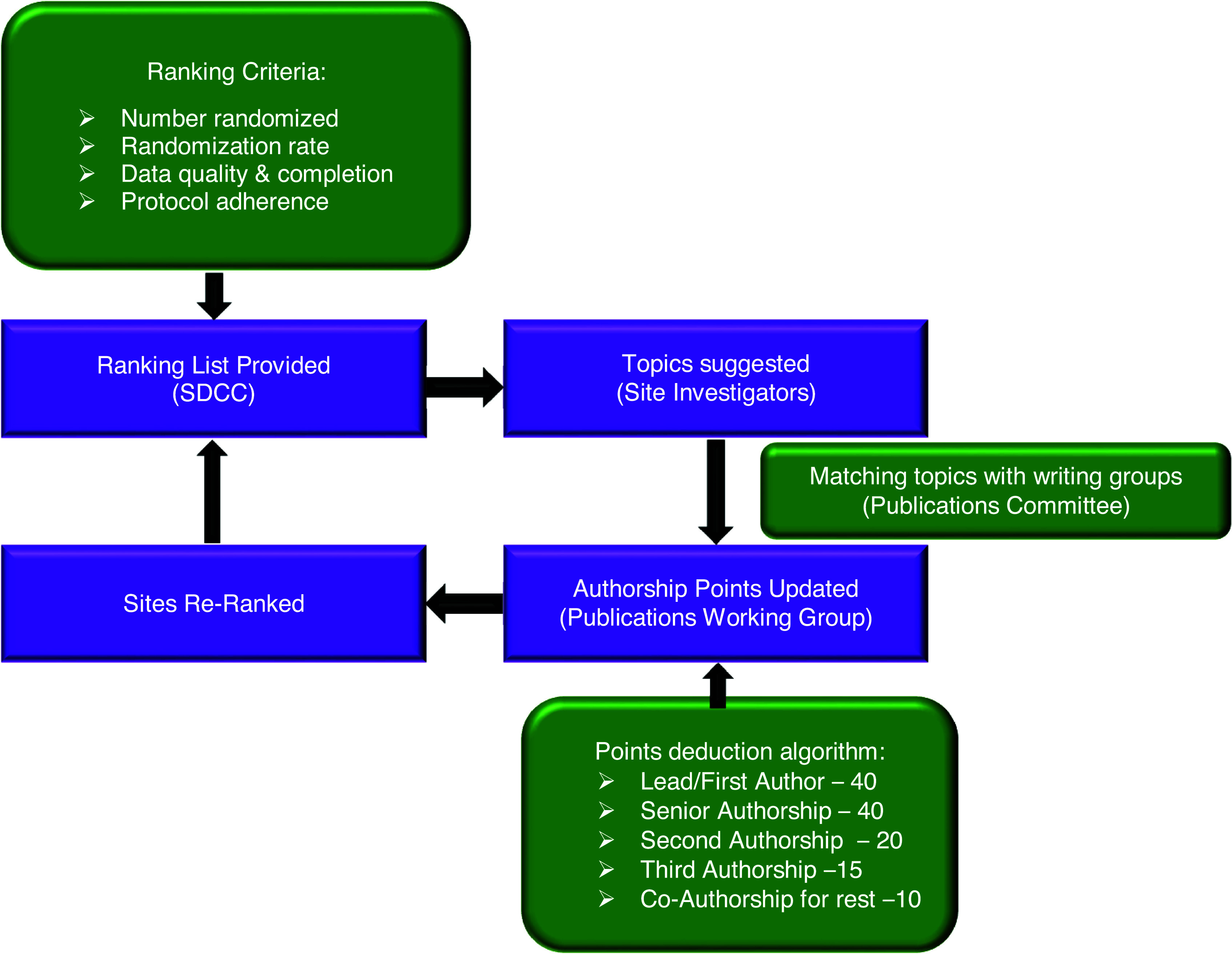
Summary of ISCHEMIA publications ranking process.

The Publications Committee and study leadership identified potential lead authors of manuscripts based on their subject matter expertise and study contribution. Prospective lead authors included country leaders, coordinating center and/or core laboratory leads, and site research team personnel. Site investigators with multiple authorship nomination slots, due to point accrual, were encouraged to share the slots with major contributors from their sites. Individual emails were sent to prospective lead authors inviting them to be the writing group lead for specific topics, and based on their response, the Publications Committee assigned a lead author and Publications Committee Liaison for the topic. Senior authorship positions were reserved primarily for the Study Chair and Co-Chair, but were also extended to leadership of the coordinating centers or committee chairs. Senior authorship positions were tracked to ensure equal distribution of senior authorship for the Study Chair and Co-Chair. The Study Chair and Co-Chair agreed on first and senior authorship for the primary trial report before trial initiation; this occurred > 10 years before publication. Certain papers were exempt from the scoring system. These included the primary ISCHEMIA study rationale and design manuscript, which was authored by the investigators who were involved in the initial grant proposal to the NHLBI. The ranking process is summarized in Figure 1.

In an effort to further ensure equitable recognition for site collaborators, we established the standardized process of including the research group name (“on behalf of the ISCHEMIA Research Group”) in the author byline of manuscripts and presentation abstracts. In 2022, we standardized the submission of a comprehensive index list of site collaborators for indexing in PubMed for each manuscript.

### Alternative paths for earning authorship and recognizing consortial contributions

Authors were invited occasionally based on the need for expertise in a given area. For each manuscript, authorship positions were reserved for important contributors to the trial who were not site investigators, including clinical and data coordinating center investigators, core laboratory directors, country leaders, steering committee members, and National Heart, Lung, and Blood Institute personnel. For example, all publications for which coronary CT angiography (CCTA) was a critical element included the CCTA core lab director and the CCTA readers. Papers that depended on stress test results included stress lab directors as authors. This approach was made transparent when describing the authorship system to all parties at investigator meetings.

Core laboratory members and individuals who had secured funding for ancillary studies were invited to lead authorship on articles related to these activities. Additional author positions on these manuscripts were assigned based upon the study’s authorship scoring system. Statisticians were recognized for their critical role in the overall study and each manuscript by having at least one dedicated contributing author position. For their analytical contributions, statisticians are often included as middle co-authors rather than lead or senior authors. ISCHEMIA’s authorship system empowered statisticians to submit their own manuscript proposals, enabling them to secure lead authorship positions.

### Site scoring system

Site investigators selected up to 10 topics of their choice and the Publications Committee matched their preferences with available spots on writing groups, depending on the site’s ranking and availability of writing group positions. When a site investigator was assigned to a writing group, points were then subtracted from the site’s score based on the authorship position. The point system for potential authorship is presented in Table 2, and the composite performance matrix for point allocation is described in Supplemental Text 2. Placement for second and/or third author was determined by the author’s contribution to drafting the manuscript. The number of publications for which Country Leaders were invited to co-author was proportional to their country/region’s contribution of participants to the trial.

Sites were re-ranked after points were spent on authorship. Sites with an inadequate number of total points for authorship were encouraged to propose topics to the Publications Committee. If approved, the proposer would be granted authorship. The scoring system and criteria for writing group invitations were presented at Steering Committee and Investigator meetings to inform attendees and to motivate site performance.

### Manuscript topics

The initial list of manuscript topics was generated by brainstorming sessions held by the Publications Committee. Those deemed highest priority were circulated to site investigators and other investigators as described above for ranking their topic preferences. If an investigator was interested in an additional topic not included on the circulated list, they had the opportunity to submit a proposal to the Publications Committee for review. Pertinent information in the proposal form included hypothesis and/or statement of intent, inclusion and exclusion criteria, data requested, primary outcomes and covariates, preliminary table shells, and a brief statistical analysis plan (see Supplemental Appendix). Proposals were generally reviewed by the Publications Chair and after incorporation of any suggested changes, the proposal was submitted to the Publications Working Group, a subset of the Publications Committee, for review. If approved, the proposer became the lead author for that writing group. After all sites used all of their points for authorship, site investigators were invited on an occasional basis to participate in writing groups depending on their area of expertise and their sites’ contribution to the trial.

### Recognizing consortial contributors

To recognize all contributors to ISCHEMIA, including site investigators, site coordinators, Clinical Coordinating Center staff, and Statistical and Data Coordinating Center staff, comprehensive, organized lists of nonauthor collaborators were created. These nonauthor collaborators are referred to as the ISCHEMIA Research Group. This list of nonauthor collaborators is submitted to scientific journals with new manuscripts for the purposes of PubMed indexing. Upon publication and indexing in PubMed, manuscripts recognizing the ISCHEMIA Research Group will appear when searching for any individual nonauthor collaborator of ISCHEMIA.

### Surveying ISCHEMIA investigators on perceptions and experiences of the authorship system

Given the lack of published literature about point-based authorship systems, we developed an online questionnaire to survey site investigators about their perceptions of and experiences with ISCHEMIA’s authorship system (see Supplementary Appendix). The survey consisted of 14 items and was comprised of 5-point Likert scale (1 – strongly disagree, 2 – disagree, 3 – neither agree or disagree, 4 – agree, 5 – strongly agree), dichotomous (yes/no), and open-ended, qualitative questions related to the system. ISCHEMIA investigators (*n* = 334) were invited via email to complete the anonymous survey. The survey was open for 2 months (from January to March 2024) and received exempt status as a quality improvement project through the Institutional Review Board (IRB) at NYU Langone Health. Data from surveys that were only partially complete were not used.

## Results

### Publications summary

A descriptive summary of ISCHEMIA publications is presented in Table 3. As of April 30, 2025, a total of 50 trial-related manuscripts had been published or were in press with authors spanning 33 countries, with an average number of 21 authors per manuscript. Fifty original, peer-reviewed manuscripts were published from the HF-ACTION trial within 6.5 years after the conclusion of study enrollment [1]. To date, 50 original, peer-reviewed ISCHEMIA trial manuscripts have been published 5.5 years after the primary results were published. To date, 96 sites have been represented in the published manuscripts, and 4 additional sites are represented in manuscripts that are presently in preparation. In ISCHEMIA, 125 site PIs and country leaders were women. A woman was lead or senior author on 24 published manuscripts, and 38 women were co-authors. While the Study Chair served as lead or senior author on 16 manuscripts, 5 other women also served as lead or senior author across 11 manuscripts. It is highly unusual for the Study Chair of such an important trial to be a woman, and this should not be minimized, but rather, recognized as an important distinction that is sadly infrequent. A more comprehensive publication summary is available in the Supplemental Appendix.

**Table 3.** Descriptive summary of ISCHEMIA publications

Number of ISCHEMIA Publications as of 10/31/2025	50
Number of Writing Groups Formed (including papers in progress)	75
Number of Trial Sites	320
Number of Sites with at Least 1 Author	96
Number of Countries with Co-Authors	31
Average Number of Authors per Manuscript	21
Number of Sites Meeting Goal of 15 Randomized Patients	63

There were some manuscripts assigned to lead authors that were not completed due to competing priorities or because the lead author did not complete the work, and other manuscripts that were reassigned to other lead authors for the same reason. Site authors who were invited to participate in one of 10 manuscripts that did not progress were reallocated to participate in other manuscripts that were in the drafting stage and were in similar topic areas. Additionally, a total of 6 in-progress and published manuscripts were assigned to another lead author, and in many cases, reassigned to a more junior investigator.

### Authorship by site

A total of 94 (29%) of the 320 sites had an author assigned to at least one ISCHEMIA manuscript (Table 1). Sites had a target randomization rate of 0.25 patients per site per month, equating to 3 patients randomized per year. Over a mean follow-up period of 5 years, sites had a target randomization rate of 15 patients. Of the 87 sites that randomized at least 15 participants over a mean of 5 years, a total of 63 sites (72%) had authorship across ISCHEMIA manuscripts.

### Results from the authorship system survey

Of 334 investigators who were sent the survey, a total of 89 (26.7%) fully completed it. Over half of respondents (61%) indicated that ISCHEMIA was the first trial they had worked on that had performance-based criteria for authorship invitation. A total of 78% of investigators agreed that the system was easy to understand. Approximately 80% agreed that the system was successful in providing a transparent method of selecting authors for manuscripts related to clinical trials. A total of 83% of respondents agreed that the system was objective, while 79% agreed that the system was fair. When asked about the system’s equitability, 58% of investigators agreed that it was equitable for early career researchers, and 57% agreed that the system was equitable for underrepresented minorities in research. Overall, 63% of those surveyed agreed that they would recommend ISCHEMIA’s authorship system to other trials. The results of the ISCHEMIA authorship system survey are presented in Table 4.

**Table 4.** ISCHEMIA authorship system survey results

Survey Item	Responses	89 (100%)
Have you been a co-author on any ISCHEMIA manuscripts?	Yes	64 (71.9%)
	No	25 (28.1%)
If you answered “yes” about being a co-author on any ISCHEMIA manuscripts, how many ISCHEMIA manuscripts have you co-authored (including papers that are still in preparation)?	1-2	34 (53.1%)
	3–5	22 (34.4%)
	6+	8 (12.5%)
Is ISCHEMIA the first trial that you worked on that had a performance-based system for selecting co-authors?	Yes	54 (60.7%)
	No	16 (18.0%)
	I did not know that ISCHEMIA had a performance-based authorship system for selecting co-authors	19 (21.4%)
How much do you know about the ISCHEMIA authorship system?	I recognize that there is an ISCHEMIA authorship system, but I do not know any of the details	31 (34.8%)
	I am not at all familiar	15 (16.8%)
	I am familiar	43 (48.3%)
It was easy for me to understand the ISCHEMIA authorship system.	Disagree	7 (7.9%)
	Neither Agree/Disagree	13 (14.6%)
	Agree	69 (77.5%)
The ISCHEMIA authorship system provided a transparent way of selecting authors for manuscripts in clinical trials.	Disagree	6 (6.7%)
	Neither Agree/Disagree	11 (12.4%)
	Agree	72 (80.9%)
The ISCHEMIA authorship system provided an objective way of selecting authors for manuscripts in clinical trials.	Disagree	4 (4.5%)
	Neither Agree/Disagree	11 (12.4%)
	Agree	74 (83.2%)
The ISCHEMIA authorship system provided a fair way of selecting authors for manuscripts in clinical trials.	Disagree	6 (6.7%)
	Neither Agree/Disagree	13 (14.6%)
	Agree	70 (78.7%)
The ISCHEMIA authorship system provided equitable authorship opportunities for early career investigators.	Disagree	7 (7.9%)
	Neither Agree/Disagree	30 (33.7%)
	Agree	62 (69.7%)
The ISCHEMIA authorship system provided equitable authorship opportunities for underrepresented investigators in medicine/science.	Disagree	6 (6.7%)
	Neither Agree/Disagree	32 (36.0%)
	Agree	51 (57.3%)
I would recommend that all clinical trials adopt this system for authorship assignments.	Disagree	4 (4.5%)
	Neither Agree/Disagree	29 (32.6%)
	Agree	56 (62.9%)

Respondents shared additional thoughts about the authorship system in response to open-ended survey questions. One investigator shared that ISCHEMIA’s authorship system “motivates young investigators (to participate in clinical research) and offers a potential way for investigators to ‘feel more invested in the program’ because of the “authorship-related recruitment incentive.” Another shared that the system was “not perfect, but it represented a pretty good attempt at providing an authorship that is fairer, objective, and merit-based when compared to most other trials;” the respondent also noted that the trial’s primary investigators were “open to listening to suggestions related to authorship from co-investigators.” In terms of opportunities for improvement, one respondent suggested that an investigator-facing “tracking system” that follows the number of points spent on particular publications and available points for forthcoming publications would be an appreciated gesture to enhance transparency.

## Discussion

The ISCHEMIA trial adapted and implemented a process for writing group invitation developed initially by HF-ACTION investigators [2,5]. Implementation of an equitable method for choosing authors based on objective trial performance metrics is of high relevance to multicenter trialists and other team science groups to reduce concerns of unfairness and bias in determining authorship. Additionally, the transparency and objectivity of our author invitation process, which evaluated contributions by each participating site, may have motivated some sites to achieve their highest level of performance.

There are key principles for success of team science that have been incorporated in “How To” documents [2,7] and publications [2,4,8–13]. These principles include effective leadership and management skills, self-awareness, trust, development of communication strategies, identification of key roles and responsibilities, and establishing how author contributions will be recognized through publications [13]. Prospective agreement on numerous aspects of study conduct is fundamental to success. As noted, when ISCHEMIA was in the early development stage, accomplished by a merger of two research teams, the Study Chair and Co-Chair agreed on their authorship positions in the primary trial report. The authorship policy we report here was conceived early on, and details were finalized before the primary report. This process required coordination between study leadership and investigators that prioritized creating a collaborative, trusting, and integrative authorship system with defined roles. There were very rare concerns expressed by site investigators. These included wishes to be included in the main results paper and a valid complaint about not being acknowledged on a manuscript in which we failed to include “on behalf of the ISCHEMIA Research Group.” That error prevented consortial contributors from being indexed in PubMed, a practice offered by most journals, and has now been addressed by including a list of consortial contributors with each new submission. By addressing issues such as author recognition throughout the study period, the ISCHEMIA team continues to adhere to key principles that facilitate collaboration and team science.

We encourage prospective use of this general approach in the early stages of multicenter study development. Each study has its own unique design, and study leadership must choose how to weigh different aspects (e.g., recruitment of women and underrepresented populations, timely submission of data) to reward those activities that are critical to trial success.

Authorship, a crucial aspect of research integrity, is relevant to many professionals within academic and scientific communities. Ensuring that intellectual contributions are properly credited is essential. ISCHEMIA’s system of authorship nomination may be helpful for other research teams who are exploring ways to define who qualifies as an author, determine authorship order, and address potential conflicts in authorship-related decisions.

This manuscript describes a transparent, merit-based authorship eligibility system adapted from the methodology used by Whellan and colleagues [5,6] and operationalized in a significantly larger multicenter trial. Our work confirms and extends the HF-ACTION authorship system, and is differentiated from HF-ACTION in 3 important ways. First, HF-ACTION was conducted at 82 regional centers (67 in the United States, 9 in Canada, and 6 in France). ISCHEMIA was conducted at 320 sites in 37 countries. In total, 137 different HF-ACTION authors were named in at least 1 publication as compared with 263 ISCHEMIA authors named in at least 1 publication. In total, 45 HF-ACTION sites had an author named to at least 1 article, as compared with 96 ISCHEMIA sites. Hence, ISCHEMIA demonstrated that the HF-ACTION point system concept was scalable to a considerably larger trial. Second, we provide the code developed for the ISCHEMIA authorship system for use and modification by other investigators (Supplemental Appendix). Third, we provide qualitative feedback from users of the system to illuminate key areas that worked well and those which could be improved. In team science, there must be an equitable process to select authors and an appropriate way to recognize the efforts of research teams. In an era when larger research teams and consortia are more common, we hope that this work will be useful to those who seek to implement effective authorship policies and foster team science.

In a survey of 89 ISCHEMIA investigators, a majority of respondents found the authorship system was easy to understand (78%), transparent (80%), objective (83%), and fair (79%). Only 58% of investigators agreed that the system was equitable for early career researchers, and 57% agreed that the system was equitable for underrepresented minorities in research.

Through their open-ended responses, investigators shared that this process increased motivation to participate in the trial due to the point allocation system and that it was a concerted effort to create a merit-based system that has not been present in other trials. Investigators also shared suggestions for improvement, such as a tracking system available for all investigators to see what publications are in process and where investigators could contribute.

### Limitations

It is important to note that while equitable, the ISCHEMIA authorship system does not alone address the low representation of minorities and women in clinical research and, therefore, authorship. The point-based authorship system was susceptible to internal biases. Sites serving socioeconomically disadvantaged populations or those with fewer resources could be systematically penalized, directly undermining equity goals. Given that for the majority of ISCHEMIA publications, the site author was the site PI, and the PI was not likely to be an early career or underrepresented minority, the fact that just 58% of investigators agreed that the system was equitable for early career researchers and 57% agreed that the system was equitable for underrepresented minorities in research, though more positive than expected, reflect systemic issues over which the trial had no control.

The coin flipping process for resolving rare authorship system ties is imperfect; when possible, co-lead authorship might offer a better solution. Further, the alternative pathway of authorship reserved for collaborators who were not site investigators was not integrated into the point-based system and thus represents a limitation of the system itself.

Lastly, it is important to note that just 89 of the 334 invited investigators (27%) participated in the online survey; this limited sample size may not be representative of all ISCHEMIA investigators and is a limitation of this study, reducing the ability for meaningful generalization and introducing nonresponse bias The authorship scoring system used to allocate authorship positions was also open to other internal biases, as points were dependent on the completeness of data, patient adherence to trial regimens, and if patients were women or from a Veteran’s Affairs hospital site. Survey responses revealed that only 48% of respondents were familiar with the system, indicating that further socialization of the system is needed.

## Conclusion

Despite the low response rate of the online survey intended to measure the successes and challenges of the ISCHEMIA authorship system, the results can still be informative for others seeking to develop their own authorship systems. The adoption and implementation of an equitable process for offering authorship opportunities to those who can meet ICMJE criteria is of high relevance to trialists and other team science groups as it reduces investigator concerns about unfairness in the authorship selection process by its focus on merit-based invitations. In addition, the transparency and objectivity of this process may motivate sites to achieve their highest level of performance.

## Supporting information

10.1017/cts.2025.10214.sm001 Esquenazi-Karonika et al. supplementary material Esquenazi-Karonika et al. supplementary material

## Supplementary material

The supplementary material for this article can be found at https://dx.doi.org/10.1017/cts.2025.10214.

## Clinical Trials.gov Identifier

NCT01471522; https://clinicaltrials.gov/ct242/show/NCT01471522
