# Supplementary material for: A point-based system to determine authorship eligibility in a large clinical trial: Insights from the ISCHEMIA trial’s authorship nomination system
Source: J Clin Transl Sci. 2025 Dec 26;10(1):e7. doi: 10.1017/cts.2025.10214 (PMC12895453; doi:10.1017/cts.2025.10214)
Supplement: Esquenazi-Karonika et al. supplementary material [file S2059866125102148sup001.docx]

Supplemental Material

Team Science in ISCHEMIA (S. Esquenazi-Karonika et al., 2025)

Contents

[Supplemental Table 1. ISCHEMIA Publication Summary 2](#_Toc212797482)

[Supplemental Material 1. ISCHEMIA Site Composite Performance Metric 5](#_Toc212797483)

[Supplemental Material 2. Authorship System Experiences and Opinions Survey 8](#_Toc212797484)

[Supplemental Material 3. ISCHEMIA Publications Standard Operation Procedures 19](#_Toc212797485)

[Supplemental Material 4: ISCHEMIA Database Research Proposal Form 28](#_Toc212797486)

[Supplemental Material 5. ISCHEMIA Publications Policy 29](#_Toc212797487)

[Appendix S1: ISCHEMIA Site Investigators 39](#_Toc212797488)

[Appendix S2: ISCHEMIA Committee, CCC, Trial-Related Personnel 105](#_Toc212797489)

# Supplemental Table 1. ISCHEMIA Publication Summary

| **#** | **Manuscript** | **Lead Author** | **Journal** | **Date** | **Citation / DOI** |
| --- | --- | --- | --- | --- | --- |
| 1 | Core Lab Stress Echo Interpretations | Kataoka | Cardiovascular Ultrasound | 12/18/2015 | <https://doi.org/10.1186/s12947-015-0043-2> |
| 2 | TID during Stress Echo | Kataoka | Echocardiography | 4/3/2016 | <https://doi.org/10.1111/echo.13222> |
| 3 | Variability in Lab Troponin Decision Level for Dx of MI | Bagai | American Heart Journal | 8/1/2017 | <https://doi.org/10.1016/j.ahj.2017.04.016> |
| 4 | ISCHEMIA Design Paper | Maron/Hochman | American Heart Journal | 7/1/2018 | [https://doi.org/10.1016/j.Am Heart J.2018.04.011](https://doi.org/10.1016/j.ahj.2018.04.011) |
| 5 | Perspective "ISCHEMIA: Establishing the Primary Endpoint" | Bangalore | Perspective for Circulation: Cardiovascular Quality and Outcomes | 5/14/2018 | <https://doi.org/10.1161/circoutcomes.118.004791> |
| 6 | Commentary: Planning and Conducting the ISCHEMIA Trial | Maron/Harrington/Hochman | Circulation | 8/9/2018 | <https://doi.org/10.1161/circulationaha.118.036904> |
| 7 | ISCHEMIA Baseline Charactersitics | Hochman | JAMA Cardiology | 2/27/2019 | <https://doi.org/10.1001/jamacardio.2019.0014> |
| 8 | OMT Predictors of LDL and SBP Goal Attainment at 1 Year | Newman | Circulation Cardiovascular Quality and Outcomes | 10/4/2019 | <https://doi.org/10.1161/CIRCOUTCOMES.119.006002> |
| 9 | Sex baseline differences in ischemia, CAD, Sx | Reynolds | JAMA Cardiology | 3/30/2020 | <https://doi.org/10.1001/jamacardio.2020.0822> |
| 10 | ISCHEMIA Main Results | Maron/Hochman | New England Journal of Medicine | 3/31/2020 | <https://www.nejm.org/doi/full/10.1056/nejmoa1915922> |
| 11 | ISCHEMIA QoL Analysis | Spertus | New England Journal of Medicine | 3/31/2020 | <https://www.nejm.org/doi/full/10.1056/NEJMoa1916370> |
| 12 | Heart Failure | Lopes | Circulation | 8/18/2020 | <https://doi.org/10.1161/circulationaha.120.050304> |
| 13 | CCTA vs. Cath Comparison | Mancini | JACC Cardiovascular Imaging | 1/13/2021 | <https://doi.org/10.1016/j.jcmg.2020.11.012> |
| 14 | ISCHEMIA MI | Chaitman | Circulation | 2/23/2021 | <https://doi.org/10.1161/circulationaha.120.047987> |
| 15 | DAOH (Days Alive Out of Hospital) | White | JAMA Cardiology | 9/1/2021 | <https://doi.org/10.1001/jamacardio.2021.1651> |
| 16 | Total and Recurrent Events | Lopez-Sendon | European Heart Journal | 9/13/2021 | <https://doi.org/10.1093/eurheartj/ehab509> |
| 17 | Outcomes by Ischemia and Anatomy | Reynolds | Circulation | 9/28/2021 | <https://doi.org/10.1161/circulationaha.120.049755> |
| 18 | Intermediate Left Main | Bangalore | Circulation: Cardiovascular Interventions | 1/7/2022 | [https://doi.org/10.1161/CIRCINTERVENTIONS.121.010925](https://urldefense.com/v3/__http:/url1282.ealerts.heart.org/ls/click?upn=KXyenyz1gNArCbZxpVtd4Mm1f2MSuGW8iHkY6wvqX3tH8cqBwJEkb2pDD0bukEDxHLEWlLJd0cmSHHGTv-2F1c1Q-3D-3DtMZV_YxCxpoge33FNHhRVcK23d3SMMuGhZElyYf76Ky89i-2FYDMFhO5C2BD1TPplT7i3SkswQNeGp2mLRP6cwSV5M81io9af-2BGkwYEweACo972Wlv1bGudGKTTSFSpSGVeVBxO9fwrr9rgCAp8dX-2Faf20-2BwchWKuddIt2oFQ5L6QIgsiKkb6mN-2BsJp2wLVJjDy4oVFVIJV6Q-2FbG190fbx28Wo7NE80d9ivdVhYD89yhKFT-2BpXa4C7mqYj37xedvzL6JWcpcuLRXN9johvIfg4NGgHSI1IqR8HcD9iUzB0dDd0zSTG1adpN1zFBkLLTvffkH0YR__;!!MXfaZl3l!IP808dnAdjxorrXVuTp79e7QaN6TQ76LRhbpeQbW405Qdqj5lZQA2mCGq5BMI1OiWx3sCeY$) |
| 19 | Mortality in ISCHEMIA | Sidhu | American Heart Journal | 2/2/2022 | [https://doi.org/10.1016/j.Am Heart J.2022.01.017](https://doi.org/10.1016/j.ahj.2022.01.017) |
| 20 | In Depth QoL | Mark | Circulation | 2/2/2022 | <https://doi.org/10.1161/circulationaha.121.057363> |
| 21 | Prediction of Left Main | Senior | Journal of the American College of Cardiology | 2/22/2022 | [10.1016/j.jacc.2021.11.052](https://doi.org/10.1016/j.jacc.2021.11.052) |
| 22 | Outcomes across range of kidney function (CKD/Main combined) eGFR | Bangalore | JAMA Cardiology | 6/29/2022 | <https://doi.org/10.1001/jamacardio.2022.1763> |
| 23 | Screening Log | Rodriguez | Clinical and Translational Science | 7/19/2022 | <https://doi.org/10.1017/cts.2022.428> |
| 24 | Adherence and Health Status | Garcia | Journal of the American College of Cardiology | 8/15/2022 | <https://doi.org/10.1016/j.jacc.2022.05.045> |
| 25 | INOCA in ISCHEMIA | Diaz/Reynolds | Journal of the American College of Cardiology | 9/13/2022 | <https://doi.org/10.1016/j.jcmg.2022.06.015> |
| 26 | ISCHEMIA vs COURAGE, BARI 2D, FAME 2 (Invasive and con) | Mavromatis | American Journal of Cardiology | 10/17/2022 | <https://doi.org/10.1016/j.amjcard.2022.09.008> |
| 27 | Health Status and Clinical Outcomes in Elderly | Ngyuen | Journal of the American College of Cardiology | 4/24/2023 | <https://doi.org/10.1016/j.jacc.2023.02.048> |
| 28 | Completeness of Revasc and Clinical Outcomes | Stone | Journal of the American College of Cardiology | 7/17/2023 | <https://doi.org/10.1016/j.jacc.2023.05.025> |
| 29 | Completeness of Revasc and QOL | Mavromatis | Journal of the American College of Cardiology | 7/17/2023 | <https://doi.org/10.1016/j.jacc.2023.06.015> |
| 30 | Biomarkers | Newman | American Heart Journal | 8/19/2023 | <https://doi.org/10.1016/j.ahj.2023.08.007> |
| 31 | Sex Differences in Clinical Outcomes | Reynolds | Journal of the American Heart Association | 3/5/2024 | <https://doi.org/10.1161/JAHA.122.029850> |
| 32 | Risk Stratification | Leipsic | JACC: Cardiovascular Imaging | 3/13/2024 | [10.1016/j.jcmg.2023.11.015](https://doi.org/10.1016/j.jcmg.2023.11.015) |
| 33 | H.T.E of Health Status | Arnold | Journal of the American College of Cardiology | 4/16/2024 | <https://doi.org/10.1016/j.jacc.2024.02.019> |
| 34 | CATH in CON | Pracon | Circulation:Cardiovascular Interventions | 4/17/2024 | <https://doi.org/10.1161/CIRCINTERVENTIONS.123.013435> |
| 35 | Exercise Capacity | Fleg | Circulation | 7/8/2024 | 10.1161/CIRCULATIONAHA.123.066980 |
| 36 | Comorbidity Burden and Biomarkers | Hamo | American Journal of Cardiology | 8/15/2024 | 10.1016/j.amjcard.2024.05.033 |
| 37 | Regional Variation | Ikemura | Circulation: Cardiovascular Quality and Outcomes | 9/20/2024 | 10.1161/CIRCOUTCOMES.123.010534 |
| 38 | Patient Decision Aid | Nguyen | Circulation: Cardiovascular Quality and Outcomes | 9/20/2024 | <https://doi.org/10.4244/EIJ-D-24-00240> |
| 39 | Atherosclerosis | Nurmohamed | European Heart Journal | 9/21/2024 | <https://doi.org/10.1093/eurheartj/ehae471> |
| 40 | ISCHEMIA-3VD | Bangalore | EuroInterventions | 10/21/2024 | 10.1161/CIRCOUTCOMES.124.010923 |
| 41 | Ischemia vs. CAD | Reynolds | Circulation: Cardiovascular Interventions | 12/17/2024 | <https://doi.org/10.1161/circinterventions.123.013743> |
| 42 | AUC | Slater | Circulation: Cardiovascular Quality and Outcomes | 3/17/2025 | 10.1161/CIRCOUTCOMES.124.010849 |
| 43 | Goal-Directed Medical Therapy | Maron/Newman | Journal of the American College of Cardiology | 4/1/2025 | [10.1016/j.jacc.2025.01.028](https://doi.org/10.1016/j.jacc.2025.01.028) |
| 44 | CTO by CCTA | Bangalore | Journal of the American College of Cardiology | 4/1/2025 | [10.1016/j.jacc.2025.01.029](https://doi.org/10.1016/j.jacc.2025.01.029) |
| 45 | CHIP | Muller | Circulation: Genomics and Precision Medicine | 4/10/2025 | 10.1161/CIRCGEN.124.004921 |
| 46 | PCI/CABG | White | American Heart Journal | 5/20/2025 | 10.1161/CIRCULATIONAHA.125.073591 |
| 47 | Stress Echocardiogram | Picard | Journal of the American Society of Echocardiography | 6/2/2025 | 10.1016/j.echo.2025.03.006 |
| 48 | Comparative Prognosis | Shaw | JACC: Cardiovascular Imaging | 9/1/2025 | 10.1016/j.jcmg.2025.03.016 |
| 49 | Health Status and PCI/CABG | Huded | Circulation | 9/5/2025 | 10.1161/CIRCULATIONAHA.125.073591 |
| 50 | Trajectories | Ikemura | Journal of the American College of Cardiology | 9/16/2025 | 10.1016/j.jacc.2025.06.044 |

# Supplemental Material 1. ISCHEMIA Site Composite Performance Metric

**Composite calculation**

Sum for each randomized participant:

+1 for all participants

+ 1/10 if participant is female and not a VA site (or + 1/40 if VA site)

- 1/4 if core lab ETT interpretation is mild or moderate or imaging interpretation is mild

- 1/2 if core lab ETT or imaging interpretation is no ischemia

- 2 if participant is non-adherent to randomized treatment strategy

- 2 if participant is full withdrawal or has no follow-up for the 2 most recent expired visit windows

-1 if participant is partial withdrawal (and not full withdrawal or missing follow-up for the 2 most recent visit windows)

- 1/2 if missing/unknown for diabetes and/or current angina and/or ejection fraction

+ 1/4 if aspirin use at last visit with non-missing aspirin data

+ 1/4 if non-smoking at last visit with non-missing smoking data

+ 1/4 if average of SBP across last 2 visits with non-missing SBP data is <140

+ 1/4 if average of LDL across last 2 visits with non-missing LDL data is <70 mg/dL

**Data definitions / notes**

**+1 for all participants**

Counted participants with a non-missing randomization date. Patients were assigned to ISCHEMIA-CKD if patient # starts in "5" and to ISCHEMIA otherwise. Participants who were transferred between sites after randomization were credited to the participant's current site.

**+ 1/10 if participant is female and not a VA site (or + 1/40 if VA site)**

Based on demog.gender.

Missing gender is imputed to male. Must have non-missing data for gender to get this credit.

VA sites are imputed to 1/4 which is approximately the trial average

**- 1/4 if core lab ETT interpretation is mild or moderate or imaging interpretation is mild**

**- 1/2 if core lab ETT or imaging interpretation is no ischemia**

Missing data imputed to moderate/severe. Sites are not penalized for missing stress interpretation data.

**- 2 if participant is non-adherent to randomized treatment strategy**

Randomized to CON:

Defined as at least 1 crossover cath (i.e. SITECATH record with NULL values in the indication fields for MI/UA, resuscitated cardiac arrest, heart failure, or failure of OMT).

Randomized to INV – main trial:

Defined as no SITECATH record with a procedure date within 90 days of randomization or SITECABG record with a procedure date within 180 days of randomization.

Randomized to INV – ISCHEMIA-CKD:

Defined as no SITECATH or SITECABG record.

**- 2 if participant is not retained to end of trial (ignored for now)**

Retention will be defined as having at least 1 visit on or after 12 months and at least 1 visit within 1 year of June 30, 2019. Will not count visits with only a national database search.

**- 2 if participant is full withdrawal or has no follow-up for the 2 most recent expired visit windows**

**-1 if participant is partial withdrawal (and not full withdrawal or missing follow-up for the 2 most recent visit windows)**

Withdrawal/overdue visits

Full withdrawal:

Defined as any VIS record where participant status is full or partial withdrawal and withdrawal consent status is 2=Full withdrawal of consent.

Partial withdrawal:

Based on patient’s most recent VIS record according to VISITID. Look for records where status is full or partial withdrawal and withdrawal consent status is 1 = Partial withdrawal only.

At least 2 overdue visits:

Patient is currently overdue for follow-up and the number of overdue visits is at least 2. A visit is considered to occur if there is a VIS record and the method(s) of follow-up include clinic visit, phone visit, medical record search, proxy, or other (do not count national database search). A visit becomes overdue when the upper limit of the visit window (defined as days from randomization) passes. If patient has a DEATH record then patient is automatically counted as being up to date for follow-up and will not lose points for overdue visits.

**- 1/2 if missing/unknown for diabetes and/or current angina and/or ejection fraction**

Counted # of participants with missing or unknown for any one or more of diabetes, current angina, and EF (categorical EF was used if continuous EF was missing).

**+ 1/4 if aspirin use at last visit with non-missing aspirin data**

Based on meds.meapasp. Follow-up visits only; not using randomization data. Non-missing aspirin defined as meds.meapyn is 0 or 1. Must have at least 1 visit with non-missing aspirin data to get credit.

**+ 1/4 if non-smoking at last visit with non-missing smoking data**

Based on pace.pacesmok. Follow-up visits only; not using randomization data. Must have at least 1 visit with non-missing smoking data to get credit.

**+ 1/4 if average of SBP across last 2 visits with non-missing SBP data is <140**

Based on medstat. msbpsys. Follow-up visits only; not using randomization data. Must have at least 1 visit with non-missing SBP to get credit.

**+ 1/4 if average of LDL across last 2 visits with non0-missing LDL data is <70 mg/dL**

Based on visitlab.lpldlcvl and visitlab.lpldlcun. Convert mmol/L to mg/dL by multiplying by 70/1.813.

Follow-up visits only; not using randomization data. Must have at least 1 visit with non-missing LDL to get credit.

**- 1/2 if participant ever has an InFORM exception > 60 days (checked 3x per year)**

Based on all exceptions report. Exceptions include late visits, missing forms, missing items, and open query. (Not counting answered queries.) Note: Exceptions status is spot checked by SDCC 3x per year in connection with ACC, ESC, AHA. If the patient ever has an exception outstanding >60 days at the time of spot checking then points are deducted. Having an exception outstanding >60 days will only cause points to be deducted if the patient has not already lost points for this criterion and the exception is still outstanding at the time of spot checking.

# Supplemental Material 2. Authorship System Experiences and Opinions Survey

**ISCHEMIA Authorship System Experiences and Opinions**

**Start of Block: Default Question Block**

General Introduction
**We invite you to complete this survey as an ISCHEMIA Investigator. The ISCHEMIA trial is a completed, global, randomized trial funded by the NHLBI.**
 
**The purpose of this brief survey is to understand your experiences with and opinions about the ISCHEMIA Authorship System. The results will be included in a table within a forthcoming manuscript which describes the ISCHEMIA Authorship System. We anticipate that it will take 5 minutes to complete this survey.**
 
**Thank you in advance for your time and input.**
 
**Judith Hochman, MD and David Maron, MD**
 

| Page Break |  |
| --- | --- |

Introduction **Below are some questions about your background as an Investigator on ISCHEMIA.**

Q1 Including ISCHEMIA, how many clinical trials have you worked on during your career?

________________________________________________________________

Q2 Have you been a co-author on any ISCHEMIA manuscripts?

- Yes (1)
- No (2)

*Skip To: Q3 If Have you been a co-author on any ISCHEMIA manuscripts? = Yes*

Q3 If you answered “yes” about being a co-author on any ISCHEMIA manuscripts, how many ISCHEMIA manuscripts have you co-authored (including papers that are still in preparation)?

________________________________________________________________

| Page Break |  |
| --- | --- |

Authorship System ?s **Next are some questions about your familiarity with the ISCHEMIA Authorship System and other authorship selection systems.**

Q4 Is ISCHEMIA the first trial that you worked on that had a performance-based system for selecting co-authors?

- Yes (1)
- No (2)
- I did not know that ISCHEMIA had an organized authorship selection process (3)

*Skip To: Q5 If Is ISCHEMIA the first trial that you worked on that had a performance-based system for selecting... = No*

Q5 If you answered "no" to the previous question, how did the other authorship systems compare with ISCHEMIA's performance-based authorship selection system? Ex: Different criteria for authorship consideration and selection, different number of authorship spots per manuscript, etc.

________________________________________________________________

| Page Break |  |
| --- | --- |

Q6 How much do you know about the ISCHEMIA Authorship System?

- I am not at all familiar with what the ISCHEMIA Authorship System is (1)
- I recognize that there is an ISCHEMIA Authorship System, but I don't know any of the details (2)
- I am a little bit familiar with the details of the ISCHEMIA Authorship System (3)
- I am pretty familiar with the details of the ISCHEMIA Authorship System (4)
- I am very familiar with the details of the ISCHEMIA Authorship System (5)

Q7 How did you find out about the ISCHEMIA Authorship System? Please select all that apply.

- An ISCHEMIA Meeting (1)
- Email (2)
- Word of Mouth (3)
- Other (4) __________________________________________________

Authorship Info ISCHEMIA's senior leadership developed a multipronged method to determine authorship in a transparent, objective, and equitable manner adapted from the methodology used by Whellan and colleagues (Whellan et al., 2009).

 In this system, each site earned points determined by the following criteria:
 1) Number of participants randomized, randomization rate, and percent of women randomized (with VA sites exempt from the latter criterion),
 2) Data quality, completion, and submission, and
 3) Adherence to the protocol (e.g., adherence to assigned strategy, achievement of medical therapy and revascularization goals, and proportion of participants that met all eligibility criteria, including stress tests that are deemed by the core labs to have at least moderate ischemia).

 The authorship categories and associated points and available spots are presented in the table below.

  

 All site investigators were surveyed to collect their preferences for writing groups by manuscript topics. Site investigators selected up to 10 topics of their choice and the Publications Committee matched their preferences with available spots on writing groups, depending on the site’s ranking and availability of writing group positions. When a site’s investigator was assigned to a writing group, points were then subtracted from the site’s score based on the authorship position. Authorship placement for second and/or third was determined based upon the author’s contribution to writing the manuscript. The number of publications for which Country Leaders were invited to co-author was proportional to their countries’ contribution of participants to the trial.

| Page Break |  |
| --- | --- |

Feelings **Below is a list of statements dealing with your general feelings about the ISCHEMIA Authorship System. Please indicate how strongly you agree or disagree with each statement.**

Q8 It was easy for me to understand the ISCHEMIA Authorship System.

- 1 - Strongly Disagree (1)
- 2 - Disagree (2)
- 3 - Neither Agree or Disagree (3)
- 4 - Agree (4)
- 5 - Strongly Agree (5)

| Page Break |  |
| --- | --- |

Q9 The ISCHEMIA Authorship System provided a **transparent** way of selecting authors for manuscripts in clinical trials.

- 1 - Strongly Disagree (1)
- 2 - Disagree (2)
- 3 - Neither Agree nor Disagree (3)
- 4 - Agree (4)
- 5 - Strongly Agree (5)

Q10 The ISCHEMIA Authorship System provided an **objective** way of selecting authors for manuscripts in clinical trials.

- 1 - Strongly Disagree (1)
- 2 - Disagree (2)
- 3 - Neither Agree nor Disagree (3)
- 4 - Agree (4)
- 5 - Strongly Agree (5)

Q11 The ISCHEMIA Authorship System provided a **fair** way of selecting authors for manuscripts in clinical trials.

- 1 - Strongly Disagree (1)
- 2- Disagree (2)
- 3 - Neither Agree nor Disagree (3)
- 4 - Agree (4)
- 5 - Strongly Agree (5)

Q12 The ISCHEMIA Authorship System provided **equitable** authorship opportunities for early career investigators.

- 1 - Strongly Disagree (1)
- 2 - Disagree (2)
- 3 - Neither agree nor Disagree (3)
- 4 - Agree (4)
- 5 - Strongly Agree (5)

Q13 The ISCHEMIA Authorship System provided **equitable** authorship opportunities for under-represented investigators in medicine/science.

- 1 - Strongly Disagree (1)
- 2 - Disagree (2)
- 3 - Neither Agree or Disagree (3)
- 4 - Agree (4)
- 5 - Strongly Agree (5)

| Page Break |  |
| --- | --- |

Q14 The ISCHEMIA Authorship System is good on paper, but didn't work in actual practice.

- 1 - Strongly Disagree (1)
- 2 - Disagree (2)
- 3 - Neither Agree or Disagree (3)
- 4 - Agree (4)
- 5 - Strongly Agree (5)

Q15 I would recommend that all clinical trials adopt this system for authorship assignments.

- 1 - Strongly Disagree (1)
- 2 - Disagree (2)
- 3 - Neither Agree or Disagree (3)
- 4 - Agree (4)
- 5 - Strongly Agree (5)

| Page Break |  |
| --- | --- |

Open ended questions **The next section asks a few open-ended questions about your overall opinion of the ISCHEMIA Authorship System. Please provide 1-2 sentence responses for each question. Note: This section is optional.**

Q16 Overall, what do you personally see as the best, most effective elements of the ISCHEMIA Authorship System?

Q17 Are there any aspects of the ISCHEMIA Authorship System that you think should be refined, improved, or changed? If so, please describe.

Q18 Is there anything else about the ISCHEMIA Authorship System that you think is important to share?

**End of Block: Default Question Block**

# Supplemental Material 3. ISCHEMIA Publications Standard Operation Procedures

**Purpose:** To outline each task and sub-task with timelines related to publication of ISCHEMIA manuscripts

**Scope:** Applicable to manuscripts planned to be published for the ISCHEMIA trials, under the purview of the ISCHEMIA Publications Committee

**Responsible Person/Unit:** ISCHEMIA Publications Committee; ISCHEMIA Publications Manager; ISCHEMIA Publications Working Group; SDCC; Biostatistician; PC Liaison; Lead Author/Writing Group Chair; co-authors of Manuscript; NHLBI

**Definitions:**

Lead Author leads analysis and drafts the contents of manuscript Writing Group Chair – assigned for each manuscript and works with the lead author to facilitate communication between co-authors, SDCC and the Publications Committee for SAP and manuscript development. A lead author can also serve as the Writing Chair and vice versa.

Publications Committee Liaison – A liaison from the Publications Committee to the writing group that helps oversee and coordinate the process from study leadership

Senior Author – the last author on a manuscript; this will usually be Study Chair or Co-Chair, or depending on the topic, may be PI of ICC, EQOLCC, or SDCC or a Committee Chair or someone else in a leadership role, approved by Chair and Co-Chair. See “Senior Author Tracker” for tracking the equal distribution of senior authorship for Study Chair and Co-Chair.

Publications Committee (PC)– committee that oversees all publications (manuscripts, abstracts) and presentations, establishes writing groups, defines manuscript priorities and deadlines as related to this SOP; composed of core members of the trial including study leadership

Publications Manager– tracks and manages timeline for trial related manuscripts, abstracts and/or presentations; corresponds with writing group for status of tasks

NHLBI – National Heart, Lung, and Blood Institute

SDCC – Statistical and Data Coordinating Center; works with lead author/writing chair to develop SAP and perform analyses

SAP – Statistical Analysis Plan; proposed by lead author, drafted by lead statistician

Publications Working Group – Internal group consists of Publications Chair, Study Chair, Ancillary Study PIs, SDCC PIs, statisticians and NHLBI officers

**Resources:**

Publications Timeline

ISCHEMIA Manual of Operations – Section 17 Publication Policy

NIH Policy

NIHMS Tracker

**Procedure:**

1) Topic Proposal by Study Leadership or Site Investigator (NA)

Study Leadership and/or Site Investigator proposes a topic for potential ISCHEMIA manuscript. A research proposal form must be submitted for review by Publications Working Group and/or Publications Committee.

2) Publications Working Group assigns a lead author / writing group chair. (NA)

Once the topic is approved, the Publications Working Group selects lead author/Writing (WG) chair, and PC Liaison. If needed, it will be sent to the Publications Committee for review and approval.

3) Lead author completes proposal form (< 2 weeks)

The lead author completes proposal form in a given time period. During this time, lead author may consult with PC Liaison who was chosen for the manuscript and has expertise in the core topics of the manuscript.

4) Feedback by PC Liaison (< 1 week)

A PC Liaison is assigned to each proposal. The Lead Author sends the first draft of the proposal to the assigned PC Liaison for review and approval.

5) Lead Author completes revisions/PC Liaison reviews & approves proposal for WG circulation (< 2 weeks)

Based on the feedback received from the PC Liaison, lead author revises the proposal and once PC Liaison approves the revision, it is circulated to the Publications Working Group for review.

6) PWG completes 1^st^ review of proposal (< 1 week)

Publications Working Group (PWG) is to review the research proposal submitted by the lead author. During this time, estimated timeline is also developed by the Publications Manager

7) PC Manager/PC Liaison provide feedback to Lead Author (< 1 week)

Any comments received from the Publications Working Group collected during this period and are sent to the lead author.

8) Lead Author completes 1st revision/PC Liaison approves revised proposal for PWG circulation (< 2 weeks)

If needed, Lead Author revises the proposal based on the feedback he/she receives from the Publications Working Group. Upon PC Liaison’s approval, a revised proposal is to be circulated to the Publications Working Group. A track change must be shown in the document.

9) WG completes 2^nd^ review of proposal (< 1 week)

The Publications Working Group reviews the revised proposal

10) PC Manager/PC Liaison provide feedback to Lead Author (< 1 week)

Same as step #7

11) Lead Author completes 2nd revision/PC Liaison approves revised proposal for WG circulation (< 1 week)

*Same as step #8*

**Step 7 - 11 are to be repeated as needed*

12) Select Writing Group (< 2 weeks)

Publications Working Group selects site investigators to serve on the writing group based on topics they requested. Inclusion criteria for site writing group members are based on sites’ performance ranking. Country Leaders and Committee members are assigned according to their contribution of the trial.

13) Send invitation and proposal/draft SAP to writing group members with RSVP deadline (< 1 week)

On behalf of PC Chair and Study Chair, the CCC sends a formal invitation to selected writing group members, requesting each member to reply to confirm their acceptance of co-authorship.

The CCC sends reminder emails to each selected member until a response is received. Reminders should be sent to non-responding co-authors (via fax, email, and phone) until they respond, in order to be considered for authorship. After the 3^rd^ official email reminder, if there is still no response, the PC Chair sends final email notification before removal of non-responding author(s). He can also seek advice from the Publications Committee to decide to move forward with the manuscript without the non-responsive member or select another member to join the writing group.

14) Writing group members RSVP, review proposal/draft SAP (< 2 weeks)

The response of the writing group members is entered into RedCap (See SOP 29-12).

**Step 12-14 concurrent with step 1-11 OR 15-25*

15) Assign Biostatistician, kick off call with to be scheduled (1day)

Once proposal is finalized, lead author submits a clean version of the proposal to the statistician and a kick off call to discuss analysis is scheduled to discuss the SAP.

16) Work on SAP . (< 2 weeks)

The assigned statistician, lead author, PC Liaison and other investigators as needed work on the development of SAP.

17) Review and either approve proposal and draft analysis plan or send back to lead author, PC liaison and statistician with comments. (< 2 weeks)

The preliminary analysis plan is circulated to the writing group for FYI.

18) Once approved by PC, statistician initiates programming (< 4-6 weeks)

19) Sign off by mentor statistician and PC Liaison (< 1 week)

Once the SAP is finalized and there aren’t any substantial changes, it is signed off by the mentor statistician assigned by the SDCC PI.

*If there are substantial changes to the proposal or analysis plan, PC Liaison notifies the Publications Committee to re-review. Additional time may be allotted for this.*

20) Perform analyses and send data to Lead Author/Writing Group Chair (< 4-6 weeks)

The SDCC performs analyses and once completed, sends data to Lead Author/PC Liaison for review.

Reminder emails are sent two weeks and one week and daily for 3 days prior to deadline. After 6 weeks, if data has not been sent to Lead Author/PC Liaison, the CCC sends a follow-up email to the SDCC on the status of analyses

21) Lead Author in collaboration with PC Liaison and writing group drafts manuscript (< 2 months)

After the data are received from the SDCC, Lead (and co-lead, if applicable) Author drafts manuscript with assistance from the assigned statistician(s), PC liaison and writing group.

During this time, the Publications Manager collects author information on affiliation, degrees, conflicts of interest that are necessary for submission of manuscript to target journal. *Any non-responsive co-authors may be removed from the writing group.* This decision will be made by the Leadership.

22) Review and/or approve draft manuscript (< 2 weeks)

The Lead Author/PC Liaison submits the manuscript draft to the writing group for review. At least two members from the Publications Committee are assigned to review, approve and/or provide feedback. (see MOO 17.4.5) Publications Team will refer to SOP 29-12 for index list preparation.

23) Revise manuscript and submit "final" draft to Publications Committee Members in the Writing Group (< 1 week)

The Lead Author/PC Liaison revises the manuscript after feedback from the writing group is received and send to the Publications Members in the writing group for another review.

24) Review and approve final draft (< 2 weeks)

The Lead Author will send the draft for administrative review to the Publications Coordinator and Manager (e.g., add ISCHEMIA Research Group byline, check COI, references, etc.). Pubs Team will send formatted draft (manuscript, supplement, index list prepared in accordance with SOP 29-12) to Lead Author, PC Liaison, and Drs. Maron and Hochman. A majority of the PC members must approve the final draft. Lead Author sends the draft for “final sign off” to leadership and faculty statistician. If an NHLBI staff is a co-author of the manuscript, it must be approved by the NHLBI before submission.

25) Submit to target journal (< 1 week)

The Publications Manager sends the order of authors to the PC Chair for review. Publications Team will ensure that indexing list is submitted as per journal specifications (SOP 29-12). After it is approved by the leadership, the Publications Manager submits the manuscript to the target journal and sends notification to the writing group.

Publications Manager files the version submitted to the journal on ICHEMIA CCC Ops Drive. Manuscript and authorship information are also entered in RedCap database and maintained by the CCC.

If manuscript is Accepted: Refer to SOP 29-07 for list of detailed tasks

If manuscript is Published

The Lead Author notifies the Publications Manager and Leadership. The Publications Manager drafts email to notify co-authors. The Publications Manager updates database (RedCap, Shared Drive) and other internal tracker(s) to reflect the approval status. (1 day within notification)

2) Publications Manager searches the journal website periodically to see if/when the paper has been published online/in print and if a reference/citation is available on the PubMed website. (1 month)

When published, Publications Manager (1) reviews the paper, figures, table and supplementary material to ensure that the final versions are used and advises lead author(s) of any discrepancies; (2) updates the PubMed link on RedCap and internal document as needed, (3) emails a PDF copy to the writing group and PC, and (4) Files a copy in the Ops Drive – Publications Folder.

3) Pub Med Central/NIH Manuscript Submission:

a. After a manuscript is accepted for publication, if the journal/ publisher does not directly send the final version to the PubMed Central/NIH Manuscript Submission (NIHMS) website, upload the final approved/accepted manuscript to the PMC/NIHMS website in compliance with the NIH Public Access Policy (<https://www.nihms.nih.gov/db/sub.cgi>). Journals that submit all NIH-funded final published articles to PubMed Central can be found at: http://publicaccess.nih.gov/submit_process_journals.htm.

b. If journal is not in above list, Publications Manager should upload final version to PMC within 12 months of publication, after checking to make sure that the journal’s designated delay period has expired.

c. When citing a paper in NIH applications, proposals, and annual progress reports, include the PMC ID at the end of the full citation. This requirement only applies to papers that fall under the Policy and are authored or co-authored by you or arose from your NIH award.

d. All published manuscripts should have the NIH PubMed Central number.

4) Check for Acknowledgement of Funding and any other required reviews (Refer to MOO Section 17.4.7)

Send final version of manuscript to donors.

If manuscript needs to be revised or is rejected and being submitted to another journal:

1) Respond to reviewer comments and revise manuscript (this may require new analyses) and send to PC (2-4 weeks)

If the manuscript needs to be revised for resubmission, Lead Author/PC Liaison, writing group and SDCC work together to respond to reviewers’ comments and send to leadership for final approval. If the manuscript is rejected from the initial journal, select a new target journal. The Publications Manager collects additional information from co-authors if needed.

2) Review and approve revised manuscript (1 week)

The Publications Committee in the writing group and NHLBI (if manuscript is considered a primary paper, major change has occurred, or NHLBI staff is a co-author) review and approve revised manuscript.

3) Incorporate comments from Publications Committee and NHLBI if any (1 week)

The lead author incorporates comments received from the PC and NHLBI for resubmission.

4) Final Approval (1 week)

The leadership reviews manuscript and gives a final approval.

5) Submit to target journal (1 week)

The Publications Manager submits manuscript to target journal and follows the process above depending on the journal’s response.

# Supplemental Material 4: ISCHEMIA Database Research Proposal Form

**A. Lead Author**

1. Lead Author’s Name *(one individual only, responsible for proposal)****:***

**B. Contact Information**

1. Lead Author’s E-mail Address *(will be listed publicly with other research proposals that are approved***):**
2. Lead Author’s Affiliated Institution**:**
3. Lead Author’s Telephone Number**:**
4. Lead Author’s Street Address**:**

**C. Areas of Research Expertise Needed for Review of Proposal**

**D. Plans for Presentation and Publication**

1. Target meeting for presentation and date
2. Target journal for submission and date

**E. Working Title** (*The title you anticipate using on your manuscript.)*

**F. Hypothesis and/or Statement of Intent 10/31/2025**

*Provide a brief statement* ***(maximum 1–2 sentences)*** *describing the proposal’s main hypothesis. Limit to no more than two aims.*

**G. Background/Significance**

*Provide a brief statement* ***(maximum 1–2 paragraphs, not more than 1 typed page)*** *describing the background and significance of the proposed research.*

**H. Inclusion & Exclusion Criteria**

*Briefly describe the proposal’s cohort inclusion and exclusion criteria. Refer to the protocol as needed to describe criteria.*

**I. Data Requested, Including Primary Outcomes and Covariates**

*Please utilize the appropriate data collection form(s) (from InForm database) as reference to delineate groups for comparison, list the primary and secondary outcomes of interest for this proposal and requests for modeling these outcomes, any covariates of interest, and any of the main variables that may need to be considered (e.g., for adjustment) in the analysis.*

**J. Brief Statistical Analysis Plan**

*Provide a brief description of the proposed statistical methodology that could be considered for your proposal based on the data requested above. Refer to "Checklist for Authors" as a reference as you develop your analysis plan. If your proposal is approved, the Statistical & Data Coordinating Center will discuss the comprehensive details of the statistical analysis plan that will support your analysis. Please provide 1-3 sample tables for how you plan to present the data.*

**K. References** (*List relevant literature citations; maximum of 10.)*

*Please return completed forms to Publications Committee.*

# Supplemental Material 5. ISCHEMIA Publications Policy

**17.0 Publication Policy**

**17.1. General Statement of Editorial Policy**

***17.1.1 Timing of Publications***

***17.1.2 Access and Participation***

***17.1.3 Restrictions on Use of Site-Specific Data***

**17.2 Responsibilities of Publications Committee**

**17.3 Definitions and Categories**

**17.4 Application, Preparation, and Submission Procedures**

***17.4.1 Submission of Proposals***

***17.4.2 Publications Committee Review and Recommendation***

***17.4.3 Formation of Writing Groups***

***17.4.4 Data Analysis***

***17.4.5 Manuscript Preparation and Approval***

***17.4.6 Authorship Policy***

***17.4.7 Acknowledgement of Federal Funding***

**17.5 Presentations and Procedures**

**17.6 Data Access and Sharing**

**17.7 Ranking Process for Publications**

**17.1 General Statement of Editorial Policy**

It is anticipated that the International Study of Comparative Health Effectiveness with Medical and Invasive Approaches (ISCHEMIA) trial will generate considerable new data relative to the evaluation and management of patients with stable ischemic heart disease.

The goal of the ISCHEMIA Publications Committee is to facilitate dissemination of the maximum amount of information from the trial in a scientifically sound and ethically responsible fashion

The procedures adopted are intended to assure: scientific integrity of the data; consistency with the study design, accurate presentation; authorship is appropriately acknowledged; the text of each publication is well written; all investigators are aware of ongoing analysis projects to avoid duplication; and publication or presentation of ISCHEMIA data does not occur without the knowledge and approval of the Publications Committees

***17.1.1 Timing of Publications***

ISCHEMIA endpoint data, or data that might jeopardize the blinding of therapy or continuation of the project, will not be released to ISCHEMIA investigators or the public until the end of the study, which will be a time deemed appropriate by the National Heart, Lung, and Blood Institute (NHLBI) Project Office and study chair

Manuscripts concerning the study's overall design, protocol, procedures, or organizational structure that do not involve endpoint data or data collected on ISCHEMIA participants may be published prior to the end of the study. Baseline or other data that will not jeopardize the continuation of the project may be published while the trial in ongoing

Research other than endpoint studies may be conducted prior to the end of the ISCHEMIA investigation and is strongly encouraged so that the maximum information can be obtained from this trial, and the methods for evaluating and analyzing secondary endpoints may be refined in preparation for later analyses. This includes ancillary studies described in detail in a separate policy document

***17.1.2 Access and Participation***

All ISCHEMIA investigators, including those that represent the multiple subspecialties at enrolling sites, Country Leaders and Country Coordinators, core laboratories, coordinating centers, committees, and the NHLBI Program Office, are permitted to develope proposals, participate in such studies as approved by the Publications Committee, and collaborate in the development and publication of research papers based on ISCHEMIA material

Non-participating investigators will have access to data as outlined in our data sharing policy (see below)

***17.1.3 Restrictions on Use of Site-Specific Data***

Presentation or publication of the results based on outcome data from a single site or a subset of sites is prohibited. Presentation and publication of economic data by different regions/health care systems is permitted with prior approval by the Publications Committee, as is exploration of potential regional differences in outcomes and treatments/treatment effect.

**17.2 Responsibilities of Publications Committee**

Set publication strategy and appropriate timelines

Avoid conflict with and/or duplication of other publications and presentations

Review and set priorities for manuscripts and presentations

Establish writing group members for each publication. Identify a chair and co-chair (if necessary) of manuscript writing groups and coordinate their formation.

Monitor progress of each paper to ensure publication in a timely fashion.

Review, help edit, and approve all analysis plans, publications, and presentations prior to submission, and enlist the assistance of appropriate outside experts as deemed appropriate

Review and approve each writing group’s choice of an appropriate journal for publication as well as alternate choices if needed

Ensure that all NHLBI policies pertaining to publications and presentations are followed

Review and approve ISCHEMIA trial press releases in conjunction with NHLBI and the Statistical and Data Coordinating Center (SDCC)

**17.3 Definitions and Categories**

The term “publication” will refer to any original scientific report, editorial, opinion paper, book chapter, or monograph submitted to a professional or scientific journal listed in Index Medicus or any popular periodical with regional, national, or international circulation that includes ISCHEMIA methods, procedures, study data or results. This definition includes print and online publications and presentations. The order of priority for ISCHEMIA resources and review by the Publications Committee will be based on the following publication categories.

*Publication Categories*

Primary/Randomized Endpoint Comparisons. We anticipate that there will be one primary manuscript addressing the trial’s primary goals and objectives. A primary quality of life manuscript and primary economics manuscript will also be included in this category. Manuscripts in this category cannot be published prior to the end of the study (see Authorship Policy below).

Other/Secondary Manuscripts. These are papers other than the primary manuscripts that utilize the database from all participating sites. Each study will be reviewed on a case-by-case basis to determine whether results may be published prior to the end of study.

Ancillary Studies. An ancillary study is an additional study independent of the parent grant. There are several types of ancillary studies including those that require collection of new data not originally planned for collection, analyzing stored biospecimens, and nested or parallel clinical trials. Ancillary studies must first be approved by the Ancillary Studies Committee. Ancillary study manuscripts will not be submitted for publication until the primary manuscript has been accepted for publication (see Ancillary Studies Policy).

Methodological/Miscellaneous. These do not fit the definition of a primary, secondary, or ancillary study report. Examples are manuscripts focused on aspects of study design or baseline study data or regional results such as economic analyses. Manuscripts concerning the study's overall design, protocol, procedures, or organizational structure that do not involve endpoint data or data collected on ISCHEMIA patients may be published prior to the end of the study. Such major publications will be developed and reviewed according to the same guidelines used for endpoint studies.

**17.4 Application, Preparation, and Submission Procedures**

***17.4.1 Submission of Proposals***

Any ISCHEMIA investigator may submit a publication proposal for consideration by the Publications Committee. Proposal forms can be downloaded from the ISCHEMIA website. Each proposal should include the following:

Background and rationale

Clear statement of objectives or research hypothesis

Brief description of study design

Brief description of data to be used

Preliminary description of the analysis plan

Proposed collaborators

Proposed sponsor

Plans for presentation and/or publication

Proposals that do not fulfill these requirements will be returned. As a courtesy, investigators may submit an abbreviated proposal to the SDCC or Clinical Coordinating Center (CCC) for the purpose of obtaining a preliminary feasibility assessment. However, a complete detailed proposal will still be required prior to formal review and approval. Investigators are encouraged to consult with a member of the Publications Committee, SDCC, and/or CCC during the development of manuscript proposals in order to ensure feasibility and to avoid overlap with ongoing analyses.

Under special circumstances non-ISCHEMIA investigators may submit proposals and/or participate in writing groups. Such circumstances include special expertise and access to other databases for pooling of data (e.g., meta-analysis).

***17.4.2 Publications Committee Review and Recommendation***

The Publications Committee will review new or substantially revised manuscript proposals based on:

Overall trial publication strategy and publication schedule

Scientific merit

Feasibility

Analytical resource availability of the SDCC

On receipt of a complete proposal, the Publications Committee will assign a publication manager at the CCC and one or more primary reviewers. The publication manager will oversee the review process and subsequently provide continuing assistance to the authors and serve as a liaison with the Publications Committee. The primary reviewers will be responsible for evaluating the proposal for scientific merit and feasibility, and making a recommendation to the full Publications Committee. The Publications Committee will assess for overlap with approved and pending proposals. In those circumstances where there is significant overlap between members of the Publications Committee and the project proposers, ad hoc reviewers may be sought to avoid conflicts of interest and ensure a fair review. As part of the review process, the proposal will be forwarded to the SDCC for comments about feasibility and an estimate of resources required. Approval of a proposal will require a simple majority of the Publications Committee. Negative decisions about publication proposals can be appealed to the ISCHEMIA Executive Committee. The publication manager or chairperson of the Publications Committee will notify the investigator, study chair, SDCC, and CCC whether the project is approved, disapproved, or whether additional information is needed before a decision can be made.

***17.4.3 Formation of Writing Groups***

When a proposal is approved, the Publications Committee (PC) will work with the publication proposer to select or endorse a chair and co-chair (if necessary) for the writing group, and will coordinate selection of its membership. The publication proposer may be asked to chair the writing group. Selection of members of the writing group will be based primarily on tangible contributions to the study and scientific, technical, and manuscript writing expertise. Site investigators will be included in writing groups, and will be selected according to the ranking process developed for this purpose (please see 17.7 Ranking Process). The publication manager assigned by the PC will continue as the principal liaison between the writing group and the PC throughout the process of writing and submitting the manuscript, and is expected to provide assistance as necessary.

There are two processes for selecting writing group members:

The PC will survey all potential writing group members (from all components of the trial organization) for their interest in proposed manuscript topics. The list of proposed manuscripts will be circulated to the highest ranked study sites. PIs or their designated site ISCHEMIA collaborator will be asked to rank manuscript topics in which they are interested. The list will also be circulated to the NHLBI project office, trial coordinating center investigators, and core laboratory investigators.

If an investigator has a publication concept that was not included in the initial list of manuscript ideas, the investigator will have the opportunity to submit a de novo publication proposal to the PC. If the PC deems that the concept warrants publication, the investigator will be appointed to that writing group and the PC will establish members of the writing group using the ranking method described below. The proposing investigator will be considered for lead authorship.

Beginning with the highest-ranked site (please see 17.7 Ranking Process), the opportunity to serve on a writing group as first, senior, or co-author will be determined by using the choice of manuscript given by that site’s PI first and then proceeding to other study personnel if available. The PI at a site can offer his or her writing group position to another qualified collaborator of their choosing at the site (e.g., co-I, study coordinator, fellow).

The authorship selection process for sites follows these rules:

Only 1 person from a site can be an author on a specific publication, but 2 or more may be considered under certain circumstances.

A site may have more than 1 publication in process at a time.

If the next manuscript, in order of preference, does not have a lead author, the PI or selecting site author will be offered the role of lead author. If all lead authorship positions are taken, the PI or selected author will be designated as the second author or a co-author following the same process that is used for lead authorship.

Where possible, assignment to a writing group will be aligned with author expertise, (e.g. site imagers included on imaging analyses)

The following manuscripts are exceptions to the above authorship selection process:

Design

Main Results

Economics

Quality of Life

Analyses using baseline data and/or methods only

Ancillary study manuscripts

***17.4.4 Data Analysis***

Once an application is approved, the SDCC will assign a biostatistician and other appropriate personnel to work with the writing group if these individuals have not been previously identified. The biostatistician will ordinarily be designated as a member of the writing group. The writing group and SDCC, with consultation from the Publications Committee, will agree on a statistical analysis plan including specific data analyses, tabulations, and displays to be provided, as well as a target date for their completion. Not all manuscripts will require SDCC involvement. Because of the routine workload at the SDCC, it will be necessary to establish priorities for data processing and analysis. Therefore, the SDCC will, as necessary, conduct analyses on database studies in the order in which they have been approved or seek guidance from the Publications Committee for determining priorities for analysis.

***17.4.5 Manuscript Preparation and Approval***

Writing can commence at any time after the writing group is designated. However, portions of the manuscript may require the SDCC’s completion of the work before writing commences. It is the responsibility of the writing group and its chair, in collaboration with the Publications Committee, to produce first and subsequent drafts within agreed upon timeframes, select initial and alternative journals (if necessary), and ensure adherence to ISCHEMIA and NHLBI policies pertaining to publications. The Publications Committee has the right to remove members and/or appoint new members to the writing group if responsibilities are not met and/or appropriate progress is not made. In the event this is required, approval of the Executive Committee must be sought.

Once the writing group has completed a final draft, at least two Publications Committee members will provide written critiques to the writing group. In circumstances where there is significant overlap between members of the Publications Committee and manuscript writing groups, ad hoc reviewers may be sought to avoid conflicts of interest and ensure a fair review. Primary and secondary reviewers may be identified for each manuscript as needed to ensure an efficient review process. Use of outside experts in the review process is encouraged if needed and especially if conflict of interest issues are deemed to be present. Manuscript reviewers should pay particular attention to the following:

Ensuring that all publications preserve the scientific integrity of the study

Correction of any factual and conceptual inaccuracies

Preparation of comments to assist writing groups in publishing papers of the highest quality and clarity

Identification and avoidance of duplication with other planned or published manuscripts

The writing group will then have an opportunity to respond to any comments and make appropriate changes. A simple majority of the Publications Committee will be required for approval of the final draft. Formal approval from NHLBI is required for any paper including NHLBI co-author(s). If there are no comments from NHLBI, the manuscript will be submitted for publication. If there are comments, the Publications Committee will assess the responses of the writing group to them and, if they are deemed satisfactory, the manuscript will be submitted.

After submission to a target journal, additional revisions to the manuscript may be required to address issues raised during peer review. Procedures for reviewing revised manuscripts after initial submission will be individualized for each project based on mutual agreement. Final revised manuscripts will be reviewed by NHLBI staff as stated above.

Following Publications Committee approval, procedures for miscellaneous manuscripts will be individualized and can often be substantially abbreviated. There is no requirement that a writing group be named for these publications. However NHLBI staff must approve final manuscripts.

***17.4.6 Authorship Policy***

Authorship recommendations will be made by the chair of the writing group in consultation with co-authors and the Publications Committee. Disputes about authorship that cannot be resolved will be referred to the Executive Committee for a final decision. In general, decisions about authorship will be based on the following criteria:

Intellectual contribution to the conception, design, and writing of the study protocol

Enrollment of subjects into the trial

Contributions to data quality (e.g., protocol compliance, missing data, etc.)

Other major contributions to the successful completion of the study (e.g., major role in statistical analysis, core lab functions, etc.)

All investigators are expected to adhere to authorship standards described in the International Committee of Medical Journal Editors Uniform Requirements for Manuscripts Submitted to Medical Journals. The requirements state that all persons designated as authors should qualify for authorship and all those who qualify should have participated sufficiently in the work to take public responsibility for appropriate portions of the content. One or more authors should take responsibility for the integrity of the work as a whole from inception to published article. Authorship credit should be based on:

Substantial contributions to conception and design, or acquisition of data, or analysis and interpretation of data

Drafting the article or revising it critically for important intellectual content

Final approval of the version to be published

Authors should meet all conditions. Additional authorship requirements imposed by individual journals will also be met, where applicable. Authorship credit will be assigned in an equitable fashion and will be commensurate with participation and effort on the individual manuscript. While Publications Committee members will have oversight of all manuscripts, committee members will appear as authors on manuscripts based solely on the basis of actual contributions to the writing of the manuscript.

For ISCHEMIA primary manuscripts, these publications will include an appendix listing all principal investigators (and, when appropriate, co-investigators) and study coordinators in ISCHEMIA. These publications will list the authors concluding with “on behalf of the ISCHEMIA Investigators" on the byline.

***17.4.7 Acknowledgment of Federal Funding***

All publications generated from ISCHEMIA data must include the statement: “The project described was supported by Award Numbers 1U01HL105907. (Other awards [note EQOL and IICC #s], ancillary study, and Clinical and Translational Science awards will be cited in addition, when applicable.) The content is solely the responsibility of the authors and does not necessarily represent the official views of the National Heart, Lung, and Blood Institute or the National Institutes of Health.”

Clinical Trial Registration: http://www.clinicaltrials.gov. Unique identifier: NCT01471522

Financial and in-kind support of all non-federal sources will be acknowledged at the end of the manuscript, when applicable

**17.5 Presentations: Policies and Procedures**

The term presentation refers to any delivery of information to scientific, professional, or public groups that does not include a full, published manuscript. This category ordinarily includes oral and poster presentations submitted initially as an abstract and talks at regional, national, and international meetings. Presentations at local institutions do not require formal approval. A presentation may be given without prior review and approval by the Publications Committee provided that the content is limited to information that is publicly available, without added interpretation or inferences. The publication manager will prepare for the ISCHEMIA website a listing of all presentations and their authors.

ISCHEMIA presentations that include any new data must be reviewed and approved by the Publications Committee. Presentation of any outcome data prior to a formal presentation of the main results at a national or international meeting is prohibited. Presentation of the results based on outcome data from a single site or a subset of sites is also prohibited. The steps involved in submission and approval of a presentation are as follows:

1. Submission of Presentation Proposal. All proposals or invitations to present ISCHEMIA data must be submitted to the Publications Committee. In general, proposals for presentation should be related to a manuscript proposal that has already been approved. Presentation proposals should include an abstract and must be submitted no later than 14 days before the abstract deadline and 14 days before the time of the presentation. For a presentation for which there is no scientific society abstract review, an abstract must still be prepared and submitted for review in the same time frame as noted above. For oral or poster abstract presentations, it is expected that there will ordinarily be a ratio of one accepted abstract to one manuscript reporting the same data set.

2. Review and Recommendation. The Publications Committee will assign a reviewer to the proposal, who will act for the entire Publications Committee. The presenter will be informed of any required or recommended revisions to the abstract within two business days of receipt. If approved, the Publications Committee will select or endorse the presenter(s) and the proposed forum for the presentation.

3. Data Retrieval, Preparation, and Analysis by the SDCC. Requests for additional data and/or analyses from the SDCC for purposes of a presentation should be unusual since these will have been approved as part of the associated manuscript. Such requests must be submitted at least 60 days in advance.

4. Notification of Acceptance. Timely notification of the Publications Committee that an abstract has been accepted for presentation is required. (Within 10 days)

5. Final Abstract Submission. If the abstract submitted initially is not the final version, the final abstract is to be submitted to the Publications Committee at least 30 days before the scheduled presentation.

6. Presentation Script (Talk Copy) and Slides Submitted. A presentation script and/or slides must be sent to the Publications Committee at least three weeks prior to the scheduled presentation.

7. Authors who do not meet these requirements may be barred from participation in other writing groups.

**17.6 Data Access and Sharing**

The Publications Committee will authorize access to study data and biospecimens. Investigators must submit a proposal requesting approval to access ISCHEMIA trial data/specimens. The ISCHEMIA trial will participate in the NHLBI Central Repository for study data and specimens. All data access will follow guidelines described in the Guidelines for NHLBI Data Set Preparation (http://www.nhlbi.nih.gov/research/funding/human-subjects/set-preparation-guidelines).

**17.7. Ranking Process for Selection to Writing Groups**

A summary measure of site performance for the purpose of participation in writing groups will be created by combining several aspects of site performance into a composite score. Domains to be included in the composite are the following:

Number randomized, randomization rate, and % women randomized (VA sites exempt from the latter criterion)

Data quality, completion, and submission

Adherence to the protocol, e.g., adherence to assigned strategy, achievement of medical therapy and revascularization goals, and proportion of participants that meet all eligibility criteria, including stress tests that are deemed by the core labs to have at least moderate ischemia.

Each site will be ranked on the basis of this final score. In the event that 2 sites have the same number of points, the one with the highest total number of randomizations (without regard to randomization rate) will have priority. If 2 or more are still tied, it will be resolved by a flip of a coin performed by the chair of the PC. After the final score, points for each site will be “spent” from the site’s overall score on the basis of authorship position on each manuscript. Points will be deducted in a 4:3:2:1 ratio for lead authorship: senior authorship: second or third author: co-authorship. Sites will be re-ranked after each assignment. As the PC moves down the list of topics, the sites with lower scores will be assigned authorship positions within the remaining writing groups. It is expected that authorship opportunities will be shared among site investigators who have contributed to the trial, as noted above. The CCC will work with site PIs to assure a fair distribution of authorship opportunities.

# Appendix S1: ISCHEMIA Site Investigators

| Country (No.Randomizations) | Investigator(s) | Study Coordinator(s) | City & State | Institution (No. Randomizations) |
| --- | --- | --- | --- | --- |
|  |  |  | (if applicable) |  |
| *United States (853) |  |  |  |  |
| Country Leader |  |  |  |  |
| David J. Maron, MD |  |  |  |  |
| Regional Leader for VA Sites | |  |  |  |
| William E. Boden, MD |  |  |  |  |
|  | Kreton Mavromatis, MD | John Doan, MD | Decatur, GA | Atlanta VA Medical Center (139) |
|  | Jason Linefsky, MD | Raven Lee, CCRP |  |  |
|  |  | Risha Patel |  |  |
|  | Todd Miller, MD | So Yang Cho | Rochester, MN | Mayo Clinic (50) |
|  |  | Susan Milbrandt |  |  |
|  |  | Dawn Shelstad |  |  |
|  | Subhash Banerjee, MD | Preeti Kamath, BDS, MHA, CCRP | Dallas, TX | V.A. North Texas Health Care System (35) |
|  |  | Ishita Tejani, BDS, MS, MSPH |  |  |
|  | Harmony R. Reynolds, MD | Stanley E. Cobos, BA | New York, NY | NYU Langone Medical Center-Bellevue Hospital (26) |
|  | Jonathan D. Newman, MD, MPH | Kirsten J. Quiles, MS |  |  |
|  | Sripal Bangalore, MD | Raven R. Dwyer, MPH |  |  |
|  | Robert  M. Donnino, MD | Dalisa Espinosa, MBS |  |  |
|  | Lawrence M. Phillips, MD |  |  |  |
|  | Muhamed Saric, MD, PhD |  |  |  |
|  | Khaled Abdul-Nour, MD | Allison Schley, BS | Detroit, MI | Henry Ford Health System (21) |
|  |  | Heather Golden |  |  |
|  | Peter H. Stone, MD | Hermine Osseni, MS | Boston, MA | Brigham & Women's Hospital, Harvard Medical School (21) |
|  |  | Charlene Wiyarand |  |  |
|  |  | Peter Douglass, BA |  |  |
|  |  | Hayley Pomeroy, BA |  |  |
|  |  | Alexandra Craft, BA |  |  |
|  |  | Bethany Harvey, BA |  |  |
|  | James J. Jang, MD | Olivia Anaya | San Jose, CA | Kaiser Permanente San Jose (18) |
|  | Gennie Yee, MD | Phoebe Goold, RN |  |  |
|  | Steven Weitz, MD | Steven Giovannone | Schenectady, NY | Cardiology Associates of Schenectady P.C. (17) |
|  |  | Lori Pritchard, RN |  |  |
|  | Suzanne Arnold, MD | Rosann Gans, RN | Kansas City, MO | Saint Luke's Hospital (17) |
|  | James Henry O’Keefe, Jr, MD (PI from 2012-2016) | Paul Kennedy, RN |  |  |
|  | Michael D. Shapiro, DO | Shobana Ganesan, PhD | Portland, OR | Oregon Health & Science University (17) |
|  |  | David Schlichting, LPN |  |  |
|  |  | Aynun Naher |  |  |
|  | Mohammad El-Hajjar, MD |  | Albany, NY | Albany Medical Center Hospital (16) |
|  | Mandeep S. Sidhu, MD, MBA |  |  |  |
|  | Steven A. Fein, MD | Wendy L. Stewart, MS |  |  |
|  | Mikhail T. Torosoff, MD, PhD | Kristin M. Salmi, BS |  |  |
|  | Radmila Lyubarova, MD |  |  |  |
|  | Sulagna Mookherjee, MD |  |  |  |
|  | Krzysztof Drzymalski, MD |  |  |  |
|  | Edward O. McFalls, MD, PhD |  | Minneapolis, MN | Minneapolis VAMC (15) |
|  | Santiago A. Garcia, MD |  |  |  |
|  | Stefan C. Bertog, MD | Debra K. Johnson, RN |  |  |
|  | Rizwan A. Siddiqui, MD | Rebekah R. Herrmann, RN |  |  |
|  | Areef Ishani, MD |  |  |  |
|  | Ronnell A. Hansen, MD |  |  |  |
|  | Michel Georges Khouri, MD | Kristine Arges | Durham, NC | Duke University Medical Center (15) |
|  |  | Melissa LeFevre |  |  |
|  |  | Jennifer Tomfohr |  |  |
|  | Jonathan L. Goldberg, MS, MD | Kimberly Ann Byrne | Cleveland, OH | Louis Stokes Cleveland Veterans Affairs Medical Center (14) |
|  |  | Taissa Zappernick |  |  |
|  | Richard Goldweit, MD | Sallie Canada | Englewood, NJ | Englewood Hospital and Medical Center (13) |
|  |  | Meghana Kakade |  |  |
|  |  | Patricia Mieses |  |  |
|  |  | Stanley E. Cobos, BA | Brooklyn, NY | NYU-HHC Woodhull Hospital (12) |
|  |  | Raven R. Dwyer, MPH |  |  |
|  | Ronny A. Cohen, MD | Dalisa Espinosa, MBS |  |  |
|  | Brooks Mirrer, MD | Kirsten J. Quiles, MS |  |  |
|  | Victor Navarro, MD | Magdalena Rantinella, BS |  |  |
|  |  | Jessica Rodriguez, BS |  |  |
|  |  | Olivia Mancilla, BS |  |  |
|  | David E. Winchester, MD, MS | Susan Stinson, RN | Gainesville, FL | Malcom Randall VAMC (11) |
|  | Marvin Kronenberg, MD | Terry Weyand | Nashville, TN | Vanderbilt University Medical Center (11) |
|  | Philip Rogal, MD | Sherron C. Crook |  |  |
|  | Christopher McFarren, MD |  |  |  |
|  | John F. Heitner, MD | Jean Ho | Brooklyn, NY | New York -Presbyterian/Brooklyn Methodist Hospital (10) |
|  |  | Saadat Khan |  |  |
|  |  | Mahmoud Mohamed |  |  |
|  | Ira M. Dauber, MD | Mary R. Soltau, RN | Littleton, CO | South Denver Cardiology Associates, P.C. (10) |
|  |  | Delsa K. Rose, RN |  |  |
|  |  | Rebecca J. Wimmer, RN |  |  |
|  |  | Kathy E. Siegel, RN |  |  |
|  |  | Susan Derbyshire |  |  |
|  | Charles Cannan, MD | Michelle Dixon | Portland, OR | Providence Heart and Vascular Institute (10) |
|  |  | Gerald Leonard |  |  |
|  | Sriram Sudarshan, MD | Ciarra Heard, LVN | Wichita Falls, TX | Wichita Falls Heart Clinic (9) |
|  |  | Viviana Gabriel, LVN |  |  |
|  |  | Sukie Desire |  |  |
|  | Puja K. Mehta, MD |  | Atlanta, GA | Emory University (9) |
|  | Michael McDaniel, MD | Fauzia Rashid, PhD |  |  |
|  | Stamatios Lerakis, MD | Senait Asier |  |  |
|  | Arshed Quyyumi, MD | Keyur Patel |  |  |
|  | Nanette K. Wenger, MD |  |  |  |
|  | Chester M. Hedgepeth, MD, PhD | Jennifer Gillis, APRN | Warwick, RI | Kent Hospital (9) |
|  | Heather Hurlburt, MD | Megan Manocchia, RN |  |  |
|  | Alan Rosen, MD | Susan Moore, RN |  |  |
|  |  | Elizabeth Congdon |  |  |
|  | Zakir Sahul, MD | Gail Brandt | Ypsilanti, MI | Michigan Heart, PC (9) |
|  |  | Nora Marchelletta |  |  |
|  |  | Kristina Wippler |  |  |
|  | David Booth, MD | Yvonne Taul, RN | Lexington, KY | University of Kentucky (8) |
|  | Steve Leung, MD | Jennifer Isaacs, MS |  |  |
|  | Ahmed Abdel-Latif, MD, PhD | Viktoria Bulkley, RN |  |  |
|  | Hassan Reda, MD | Caroline Rodgers |  |  |
|  | Khaled Ziada, MD |  |  |  |
|  | Sampoornima Setty, MD | Kimberly E. Halverson, RHIT | La Crosse, WI | Gundersen Lutheran Medical Center (8) |
|  |  | Christine Roraff, RN |  |  |
|  |  | Jonean Thorsen, RN |  |  |
|  | Rajat S. Barua, MD, PhD | Amarachi Ojajuni | Kansas City, MO | Kansas City VA Medical Center (8) |
|  |  | Oni Olurinde |  |  |
|  |  | Kamalakar Surineni |  |  |
|  | Fadi Hage, MD | Badhma Valaiyapathi, MD | Birmingham, AL | UAB Vascular Biology and Hypertension Program (8) |
|  | Christiano Caldeira, MD |  |  |  |
|  | James E. Davies, MD |  |  |  |
|  | Massoud Leesar, MD |  |  |  |
|  | Jaekyeong Heo, MD |  |  |  |
|  | Amy Iskandrian, MD |  |  |  |
|  | Firas Al Solaiman, MD |  |  |  |
|  | Satinder Singh, MD |  |  |  |
|  | Khaled Dajani, MD | Carol M. Kartje, BSN | Maywood, IL | Loyola University Medical Center (8) |
|  | Mohammad El-Hajjar, MD |  | Albany, NY | Samuel Stratton VA Medical Center of Albany NY (7) |
|  | Paul Der Mesropian, MD |  |  |  |
|  | Joseph Sacco, MD | Michele Rawlins, NP |  |  |
|  | Brian McCandless, MD | Jennifer Thomson, MA |  |  |
|  | Marisa Orgera, MD |  |  |  |
|  | Mandeep S. Sidhu, MD, MBA (2012-2016 ) |  |  |  |
|  |  | Mary Colleen Rogge, RN | Cincinnati, OH | Cincinnati VA Medical Center (7) |
|  | Imran Arif, MD | Julie Bunke , BA |  |  |
|  | Hanan Kerr, MD | Kendra Unterbrink , PA |  |  |
|  |  | Jacqueline Fannon, RN |  |  |
|  |  | Cynthia Burman, NP |  |  |
|  | Jorge F. Trejo (Gutierrez), MD | Marcia F. Dubin, CCRP | Jacksonville, FL | Mayo Clinic Florida (7) |
|  | Gerald Fletcher, MD |  |  |  |
|  | Gary E. Lane, MD |  |  |  |
|  | Lynn M. Neeson, DNP |  |  |  |
|  | Pragnesh P. Parikh, MD |  |  |  |
|  | Peter M. Pollak, MD |  |  |  |
|  | Brian P. Shapiro, MD |  |  |  |
|  | Kevin Landolfo, MD |  |  |  |
|  | Anthony Gemignani, MD | Sarah Beaudry, RN | White River Junction, VT | VAMC-White River Junction (7) |
|  | Daniel O'Rourke, MD |  |  |  |
|  | Judith L. Meadows, MD | Stephanie A. Tirado, RN | West Haven, CT | VA Connecticut Healthcare System (7) |
|  |  | Janet Halliday |  |  |
|  |  | Pamela Julian |  |  |
|  | Jason T. Call, MD | Stephanie, M. Lane, RN, BSN, CCRN | Winchester, VA | Winchester Cardiology and Vascular Medicine, PC (7) |
|  |  | Jennifer L. Stanford, RN, MSN |  |  |
|  | Joseph Hannan, MD |  | Worcester, MA | Saint Vincent Hospital at Worcester Medical Center (7) |
|  | Robert Bojar, MD | Patricia Arsenault, RN |  |  |
|  | Deepti Kumar, MD | Pamela Sigel, RN |  |  |
|  | John Mukai, MD |  |  |  |
|  | Edward T. Martin, MS, MD | Miriam Brooks | Tulsa, OK | Oklahoma Heart Institute (7) |
|  | Gabriel Vorobiof, MD | Ladda Douangvila | Los Angeles, CA | Ronald Reagan UCLA Medical Center (7) |
|  |  | Rubine Gevorgyan |  |  |
|  | Alec Moorman, MD | Fatima Ranjbaran, RN | Seattle, WA | University of Washington Medical Center (7) |
|  |  | Bryn Smith, BS |  |  |
|  |  | Carly Ohmart |  |  |
|  | Scott Kinlay, MBBS, PhD |  | West Roxbury, MA | VA Boston Healthcare System (6) |
|  | Robert J. Hamburger, MD |  |  |  |
|  | Thomas P. Rocco, MD | Samantha Ly, MA |  |  |
|  | Deepak L. Bhatt, MD, MPH | Margot C. Quinn, BA |  |  |
|  | Kevin Croce, MD, PhD | Sara Temiyasathit, PhD |  |  |
|  | Jacquelyn A Quin, MD | Jacquelyn Do, MPH |  |  |
|  | Jati Anumpa, MD | Desiree Tobin, MPH |  |  |
|  | Marco Zenati, MD, MSc |  |  |  |
|  | David P Faxon, MD |  |  |  |
|  | Glenn Rayos, MD | Jennifer Langdon | Daytona Beach, FL | Daytona Heart Group (6) |
|  |  | Marcia Werner Bayer |  |  |
|  | Ashraf Seedhom, MD | Amanda O'Malley | Albany, NY | Capital Cardiology Associates (6) |
|  | Lance Sullenberger, MD | Erin Orvis |  |  |
|  | Gregory Kumkumian, MD | Mandy Murphy, RN | Bethesda, MD | NIH Heart Center at Suburban Hospital (6) |
|  |  | Ann Greenberg, RN |  |  |
|  |  | Margaret Iraola, RN |  |  |
|  | Steven P. Sedlis, MD | Leandro C.Maranan, CCRC | New York, NY | VA New York Harbor Health Care System (6) |
|  | Robert M. Donnino, MD |  |  |  |
|  | Jeffrey Lorin, MD |  |  |  |
|  | Jacqueline E. Tamis-Holland, MD | Ammy Malinay, RN | Ridgewood, NJ | Mount Sinai Saint Luke's Hospital (6) |
|  | Robert Kornberg, MD |  |  |  |
|  | Robert Leber, MD |  |  |  |
|  | Souheil Saba, MD | Candice P. Edillo, RN | Southfield, MI | Providence - Providence Park Hospital (6) |
|  | Michael W. Lee, MD |  |  |  |
|  | Delano R. Small, MD |  |  |  |
|  | Wassim Nona, MD |  |  |  |
|  | Patrick B. Alexander, MD |  |  |  |
|  | Iram Rehman, MD |  |  |  |
|  | Umesh Badami, MD | Ann Ostrander, RN | Saginaw, MI | Covenant Medical Center, Inc. (5) |
|  |  | Stephanie Wasmiller, RN |  |  |
|  | Kevin Marzo, MD | Wendy Drewes, RN | Mineola, NY | NYU Winthrop (5) |
|  |  | Dipti Patel, RN |  |  |
|  | Inga H. Robbins, MD |  | Pomona, NJ | AtlantiCare Regional Medical Center (5) |
|  | Howard A. Levite, MD | Jackie M White, RN, BSN CCRC |  |  |
|  | Sanjay Shetty, MD | Alison Hallam |  |  |
|  | Mayuri Patel, MD |  |  |  |
|  | Glenn S. Hamroff, MD | Benjamin J Spooner, RPA-C | Cortlandt Manor, NY | NYP Medical Medical Group Hudson Valley Cardiology (5) |
|  |  | Linda M Hollenweger, LPN,CCRC |  |  |
|  | Raymond W. Little, MD | Holly Little | Houston, TX | Houston Heart & Vascular Associates (5) |
|  | Brandi D. Zimbelman, FNP-C | Tiffany Little |  |  |
|  | Charles Y. Lui, MD | Nona A Eskelson, RN | Salt Lake City, UT | Salt Lake City VA Medical Center (4) |
|  | Brigham R. Smith, MD |  |  |  |
|  | Daniel P. Vezina, MD, MSC |  |  |  |
|  | Lillian L. Khor, MBBCh, MSc |  |  |  |
|  | Josephine D. Abraham, MD, MPH |  |  |  |
|  | David A. Bull, MD |  |  |  |
|  | Stephen H. McKellar, MD, MSc |  |  |  |
|  | David Booth, MD | Yvonne Taul, RN | Lexington, KY | Lexington VA Medical Center (4) |
|  | John Kotter, MD | Caroline Rodgers, RN |  |  |
|  | Ahmed Abdel-Latif, MD, PhD | Jennifer Isaacs, MS |  |  |
|  |  | Viktoria Bulkley |  |  |
|  | Bob Hu, MD | Renee Kaneshiro | Palo Alto, CA | Palo Alto Medical Foundation Research Institute (4) |
|  | Arthur J. Labovitz, MD |  | Tampa, FL | University of South Florida (4) |
|  | Michael Berlowitz, MD | Bonnie J. Kirby, RN, MSN |  |  |
|  | Philip Rogal, MD | Nhi N. Tran, MS |  |  |
|  | Christopher McFarren, MD | Catherine Jahrsdorfer, RN, BSN |  |  |
|  | Fadi Matar, MD |  |  |  |
|  | Christiano Caldeira, MD |  |  |  |
|  | David J. Maron, MD |  | Stanford, CA | Stanford University School of Medicine (4) |
|  | Fatima Rodriguez, MD, MPH | Reem Yunis, PhD |  |  |
|  | Ingela Schnittger, MD | Jhina Patro |  |  |
|  | William F. Fearon, MD |  |  |  |
|  | Prakash Deedwania, MD | Antonia Vega | Fresno, CA | UCSF - Fresno Community Regional Medical Center (4) |
|  | Kiran Reddy, MD |  |  |  |
|  | Joseph Sweeny, MD | Hugo Bloise-Adames | New York, NY | Icahn School of Medicine at Mount Sinai (4) |
|  |  | Santa Jimenez |  |  |
|  |  | Nicole Saint Vrestil |  |  |
|  |  | Reyna Bhandari |  |  |
|  | Christopher Spizzieri, MD | Danielle Schade | Camp Hill, PA | Holy Spirit Hospital Cardiovascular Institute (4) |
|  |  | Roxanne Yost |  |  |
|  | Claudia P Hochberg, MD | Paula Beardsley | Boston, MA | Boston Medical Center (4) |
|  |  | Denise Fine |  |  |
|  | William D. Salerno, MD | Jana Tancredi, RN, MA/MSN, CCRN | Saddle Brook, NJ | Hackensack University Medical Center (4) |
|  |  | Patricia Arakelian |  |  |
|  |  | Susan Mathus |  |  |
|  |  | Deborah O'Neill |  |  |
|  | Ray Wyman, MD | Joy Burkhardt, CCRP | Torrance, CA | Torrance Memorial Medical Center (4) |
|  |  | Suellen Hosino, RN, BSN, CCRP |  |  |
|  |  |  |  |  |
|  |  | Oksana A. Lubyanaya, BA | Santa Ana, CA | Coastal Heart Medical Group (4) |
|  |  | Jose D. Salas, BS |  |  |
|  | Amer Zarka, MD | Maria Aguirre |  |  |
|  | Anil V. Shah, MD | Manu Dhawan |  |  |
|  |  | Diana Parra |  |  |
|  |  | Tri Tran |  |  |
|  | Thomas Haldis, DO | Catherine Weick, BSRT(R)(VI) | Fargo, ND | Sanford Health (4) |
|  |  | Katie Fowler-Lehman, BSN |  |  |
|  |  | Natalie Spitzer, BSN |  |  |
|  |  | Casey Riedberger |  |  |
|  |  | Catherine Weick |  |  |
|  | Jeffrey A. Kohn, MD | Stanley E. Cobos, BA | New York, NY | NYU New York Medical Associates (4) |
|  |  | Raven R. Dwyer, MPH |  |  |
|  |  | Dalisa Espinosa, MBS |  |  |
|  |  | Kirsten J. Quiles, MS |  |  |
|  | Saket Girotra, MD | Carrie Drum, RN | Iowa City, IA | University of Iowa Hospitals and Clinics (4) |
|  |  | Kimberly Miller-Cox, RN |  |  |
|  |  | Amy Ollinger, RN |  |  |
|  | Omar Almousalli, MD | Elizabeth Capasso-Gulve | Fairview Heights, IL | Advanced Heart Care Group (4) |
|  |  | Alaine Melanie Loehr |  |  |
|  |  | Marlowe Mosley |  |  |
|  | Mayil S. Krishnam, MD | Shirin Heydari, MS | Orange, CA | University of California Irvine Medical Center (3) |
|  | Jeffrey C. Milliken, MD | Andrea M. Lundeen, MA |  |  |
|  | Pranav M. Patel, MD | Edgar Karanjah, MD |  |  |
|  | Arnold H. Seto, MD | Wanda C. Marfori, MD |  |  |
|  | Kevin T. Harley, MD | Eduardo Hernandez-Rangel, MD |  |  |
|  | Michael A. Gibson, MD | Pam Singh |  |  |
|  | Byron J. Allen, MD |  |  |  |
|  | Rita Coram, MD | Anne Marie Webb, BSN | Louisville, KY | University of Louisville (3) |
|  |  | Ellie Fridell, BS |  |  |
|  |  | Heidi Wilson, BS |  |  |
|  | Sabu Thomas, MD, MSc | Angela Kim, BS | Rochester, NY | University of Rochester (3) |
|  | Ronald G Schwartz, MD, MS | Patrick Wilmot, BS |  |  |
|  | Wei Chen, MD, MS |  |  |  |
|  | Mahfouz El Shahawy, MD | Ramona Stevens | Sarasota, FL | Cardiovascular Center of Sarasota (3) |
|  | James Stafford, MD | Loriane Black | Baltimore, MD | University of Maryland Medical Center (3) |
|  | William B. Abernethy, MD | Amber B. Hull, RN | Asheville, NC | Asheville Cardiology Associates (3) |
|  |  | Olivia J. Lim, RN |  |  |
|  |  | Helen C. Tucker |  |  |
|  |  | Natasha C. Putnam, RN |  |  |
|  |  | Linda L. Hall |  |  |
|  |  | Tia Cauthren |  |  |
|  |  | Trish Tucker |  |  |
|  | Andrew Zurick, MD | Hollie Horton | Nashville, TN | Saint Thomas Hospital (3) |
|  |  | Jan Orga |  |  |
|  | Thomas M. Meyer, MD | Joyce R. White, MSN NP-C | Lynchburg, VA | Stroobants Cardiovascular Center (3) |
|  | Ronald G. Morford, MD | Cynthia Baumann, RN |  |  |
|  | Bruce Rutkin, MD | Vidya Seeratan | Manhasset, NY | Northwell Health - Manhasset (3) |
|  | Sabahat Bokhari, MD | Magnolia Jimenez | New York, NY | Columbia University Medical Center (3) |
|  | Seth I. Sokol, MD | Cidney Schultz, RN | Bronx, NY | Jacobi Medical Center (3) |
|  | Jay Meisner, MD | Jeanne Russo, RN |  |  |
|  | Ihab Hamzeh, MD |  | Houston, TX | Baylor College of Medicine (3) |
|  | Arunima Misra, MD | Zohra Huda, RN, BSN, CCRP |  |  |
|  | Matthew Wall Jr., MD | Araceli Boan |  |  |
|  | Veronica Lenges De Rosen, MD |  |  |  |
|  | Mahboob Alam, MD |  |  |  |
|  | Michael C. Turner, MD | Christine R Hinton | Lake Charles, LA | Cardiovascular Specialists of Southwest Louisiana (3) |
|  | Thomas J. Mulhearn, MD |  |  |  |
|  | Arnold P. Good, MD | Beth A. Archer, BSN, RN | Columbus, OH | Ohio Health Grant Medical Center (3) |
|  |  | Julia S. Dionne, BA |  |  |
|  |  | Cheryl A. Allardyce, BSN, RN |  |  |
|  |  | Lindsey N. Sikora, BSN, RN |  |  |
|  |  | Jennifer H. Czerniak, RN |  |  |
|  |  | Jennifer A. Mull, MSN, RN |  |  |
|  |  | Elizabeth Ferguson |  |  |
|  |  | Frances Laube |  |  |
|  | Nicolas W. Shammas, MD, MS | Gail A Shammas, BSN, RN | Davenport, IA | Midwest Cardiovascular Research Foundation (3) |
|  |  | Lori Christensen |  |  |
|  |  | Holly Park |  |  |
|  | Robert Chilton, MD | Joan Hecht | San Antonio, TX | Audie Murphy V.A. (2) |
|  | Patricia K. Nguyen, MD | Davis Vo, BS | Palo Alto, CA | VA Palo Alto Healthcare System (2) |
|  |  | James Hirsch |  |  |
|  | Matthew Jezior, MD | Jody Bindeman | Bethesda, MD | Walter Reed National Military Medical Center (2) |
|  |  | Sara Salkind |  |  |
|  |  | Dalisa Espinosa, MBS | Providence, RI | Miriam Hospital (2) |
|  |  | Lori-Ann Desimone, BSN |  |  |
|  | Paul C. Gordon, MD | Lina Felix-Stern |  |  |
|  | Thomas Crain, MD | Jassira Gomes |  |  |
|  |  | Catherine Gordon, BSN |  |  |
|  | Robert Stenberg, MD | Aimee Mann | Johnstown, PA | Conemaugh Valley Memorial Hospital (2) |
|  |  | Theresa McCreary |  |  |
|  | Ronald P. Pedalino, MD | Stanley E. Cobos, BA | Brooklyn, NY | NYU-HHC Kings County Hospital Center (2) |
|  |  | Raven R. Dwyer, MPH |  |  |
|  |  | Dalisa Espinosa, MBS |  |  |
|  |  | Kirsten J. Quiles, MS |  |  |
|  | Joseph Wiesel, MD | Stanley E. Cobos, BA | Flushing, NY | New York University - Langone Cardiovascular Associates (2) |
|  |  | Raven R. Dwyer, MPH |  |  |
|  |  | Dalisa Espinosa, MBS |  |  |
|  |  | Kirsten J. Quiles, MS |  |  |
|  | George J. Juang, MD | Candace Gopaul, BS | Brooklyn, NY | Coney Island Hospital (2) |
|  |  | Karen Hultberg |  |  |
|  |  | Tauqir Huk |  |  |
|  |  | Afshan Hussain |  |  |
|  | Mohammed Al-Amoodi, MD | Yesenia Zambrano, BS | Yuma, AZ | Yuma Regional Medical Center (2) |
|  |  | Sarah Medina Rodriguez |  |  |
|  |  | Trudie Milner |  |  |
|  | David Wohns, MD | Abbey Mulder, RN | Grand Rapids, MI | Spectrum Health (2) |
|  |  | Stacie Van Oosterhout, MEd |  |  |
|  | Ellis W. Lader, MD | Martha Meyer, RN, MSN | Kingston, NY | Mid Valley Cardiology (1) |
|  | Michael Mumma, MD | Nancy L. Clapp, RN, BA, CCRC | Sarasota, FL | Sarasota Memorial Hospital (1) |
|  |  | Heather Barrentine |  |  |
|  | Lekshmi Dharmarajan , MD | Jenne M. Jose, PA | Bronx, NY | NYU-HHC Lincoln Medical and Mental Health Center (1) |
|  |  | Stanley E. Cobos, BA |  |  |
|  |  | Raven R. Dwyer, MPH |  |  |
|  |  | Dalisa Espinosa, MBS |  |  |
|  |  | Kirsten J. Quiles, MS |  |  |
|  |  | Jenne Manchery |  |  |
|  | Joseph F.X. McGarvey Jr, MD | Vera McKinney, RN | Doylestown, PA | Doylestown Health Cardiology (1) |
|  |  | Linda Schwarz, RN |  |  |
|  | Thomas R. Downes, MD (till Dec. 2016) | Scott M. Kaczkowski | Loveland, CO | Medical Center of the Rockies (1) |
|  | Gary J. Luckasen, MD (from Dec. 2016) | Adam J. Jaskowiak |  |  |
|  |  | Joel Klitch |  |  |
|  | Benjamin Cheong, MD | Debra Dees | Houston, TX | Baylor St. Luke's Medical Center (1) |
|  | Srinivasa Potluri, MD | Precilia Vasquez | Plano, TX | Baylor Research Institute at Legacy Heart Center (1) ** |
|  | Ronald A. Mastouri, MD |  | Indianapolis, IN | Indiana University/Krannert Institute of Cardiology (1) |
|  | Jeffery A. Breall, MD, PhD | Elise L. Hannemann, RN,CCRC |  |  |
|  | George E. Revtyak, MD | Judy Mae Foltz, RN,CCRC |  |  |
|  | Jonathan W. Bazeley, MD |  |  |  |
|  | Dayuan Li, MD | Emily DeRosa | St. Paul, MN | HealthEast Saint Joseph's Hospital (1) |
|  |  | Beth Jorgenson |  |  |
|  |  | Joyce Riestenberg-Smith |  |  |
|  | Kenneth Giedd, MD |  | New York, NY | Beth Israel Medical Center (1) |
|  | Wayne Old, MD | Rebecca Bariciano | Chesapeake, VA | Cardiovascular Associates, Ltd. (1) |
|  | Francis Burt, MD |  | Bethlehem, PA | Saint Luke's Hospital and Health Network (1) |
|  | Kozhaya Sokhon, MD | Jessica Waldron | Sugar land, TX | Medicus Alliance Clinical Research Org., Inc. (1) |
|  |  | Michelle Mayon |  |  |
|  | Deepika Gopal, MD |  | Plano, TX | The Heart Hospital Baylor (1) |
|  | Uma S. Valeti, MD | Gretchen Ann Peichel, RN | Minneapolis, MN | University of Minnesota (1) |
|  | Jon Kobashigawa, MD | Brandy Starks | Beverly Hills, CA | Cedars Sinai Medical Center (1) |
|  |  | Lucilla Garcia |  |  |
|  |  | Maria Thottam |  |  |
| India (941) |  |  |  |  |
| Country Leader |  |  |  |  |
| Balram Bhargava, DM |  |  |  |  |
|  |  | Anjali Anand, MSc | Calicut | Government Medical College (208) |
|  | Sajeev Chakanalil Govindan, MD, DNB, DM, PhD | Janitha Raj, B.Tech |  |  |
|  | Rajesh Gopalan Nair, MD, DNB, DM | Reshma Ravindran, MSc |  |  |
|  |  | Rajalekshmi VS, MSc, MScCRRA |  |  |
|  | Cholenahally Nanjappa Manjunath, MD, DM | Nandita Nataraj, BE(Biotech) PGDICRCDM | Bengaluru | Sri Jayadeva Institute of Cardiovascular Sciences and Research (149) |
|  | Nagaraja Moorthy, MD, DM | Soundarya Nayak, BE(Biotech) PGDICRCDM | |  |
|  | Satvic Cholenahally Manjunath, MD,DM | Mahevamma Mylarappa, GNM (General Nursing) | |  |
|  | Suryaprakash Narayanappa, MBBS |  |  |  |
|  | Neeraj Pandit, MD, DM | Sheromani Bajaj | New Delhi | Dr Ram Manohar Lohia Hospital (101) |
|  | Ranjit Kumar Nath, MD, DM | Vandana Yadav, Msc,PGDACR |  |  |
|  |  | Girish Mishra, Msc, PGDACR |  |  |
|  | S.K. Dwivedi, DM | Roma Tewari, PG | Lucknow | King George's Medical University, Department of Cardiology (100) |
|  | V.S. Narain, DM | Meenakshi Mishra, PG |  |  |
|  | Sharad Chandra, DM | Shivali Patel |  |  |
|  |  | Suman Singh, PG |  |  |
|  | Gurpreet S. Wander, DM |  | Ludhiana | Hero DMC Heart Institute, Dayanand Medical College and Hospital (83) |
|  | Rohit Tandon, MD |  |  |  |
|  | Sarju Ralhan, M.Ch (CTVS) | Baljeet Kaur, MSc (Biotechnology) |  |  |
|  | Naved Aslam, DM | Sonika Gupta , MBA, B. Pharmacy |  |  |
|  | Abhishek Goyal, DM |  |  |  |
|  | Balram Bhargava, DM | Chandini Suvarna, BDS | New Delhi | All India Institute Of Medical Sciences (67) |
|  | G.Karthikeyan, DM |  |  |  |
|  | S.Ramakrishnan, DM |  |  |  |
|  | Sandeep Seth, DM |  |  |  |
|  | Rakesh Yadav, DM |  |  |  |
|  | Sandeep Singh, DM |  |  |  |
|  | Ambuj Roy, DM |  |  |  |
|  | Neeraj Parakh, DM |  |  |  |
|  | Sunil Kumar Verma, DM |  |  |  |
|  | Rajiv Narang, DM |  |  |  |
|  | Sundeep Mishra, DM |  |  |  |
|  | Nitish Naik, DM |  |  |  |
|  | Gautam Sharma, DM |  |  |  |
|  | Shiv Kumar Choudhary, M.Ch |  |  |  |
|  | Chetan Patel, DNB |  |  |  |
|  | Gurpreet Gulati, MD |  |  |  |
|  | Sanjeev Sharma, MD |  |  |  |
|  | V K Bahl, DM |  |  |  |
|  | Anoop Mathew, MD | Binoy Mannekkattukudy Kurian | Kolenchery | MOSC Medical College Hospital (39) |
|  | Eapen Punnoose, MD |  |  |  |
|  | Milind Avdhoot Gadkari, MD | Sheetal Rupesh Karwa, BHMS | Pune | KEM Hospital Pune (35) |
|  | Siddharth Gadage, MD DNB | Suvarna Kolhe, MSc |  |  |
|  | Tapan Umesh Pillay, BHMS MSc |  |  |  |
|  | Santhosh Satheesh, MBBS, MD, DM | R. J. Vindhya, B.Sc. (Bio-Technology), MSc(Bio-Informatics) | Pondicherry | Jawaharlal Institute of Postgraduate Medical Education & Research (JIPMER) (31) |
|  |  | Peeyush Jain, MD | New Delhi | Fortis Escort Heart Institute |
|  |  | Ashok Seth, MD |  | -31 |
|  |  | Zile Singh Meharwal, MD |  |  |
|  | Atul Mathur, MD | Atul Verma, MD |  |  |
|  | Upendra Kaul, MD | Mona Bhatia, MD |  |  |
|  |  | Ankush Sachdeva, MD |  |  |
|  |  | Thounaojam Indira Devi, RN |  |  |
|  |  | Nungshi Jungla, RN |  |  |
|  | Johann Christopher, MD, DNB | K. Manjula Rani, MSc. | Hyderabad | Gurunanak CARE Hospital (27) |
|  | Rajeev Menon, MD, DNB | M. Sowjanya Reddy, BSc |  |  |
|  | Nirmal Kumar, MD, DNB | K. Preethi, BSc |  |  |
|  | Abraham Oomman, MD,DM,DNB | Rinu R sidh, MSc(Clinical Research) | Chennai | Apollo Research and Innovation (23) |
|  | Robert Mao, MD, DM | Ramakrishnan T., B.Tech(Biotechnology) | |  |
|  | Hilda Solomon, PhD | Rajesh Francis, MSc(Clinical Research) | |  |
|  | Sudhir Naik, MD, DM | Vamshi Priya P., MSc | Hyderabad | Apollo Research & Innovations (13) |
|  | Sajeeda Parveen Khan, MBBS, (Dip.Card) |  |  |  |
|  | Johann Christopher, MD | Kotiboinna Preethi | Hyderabad | CARE Nampally (11) |
|  | Nirmal Kumar, MD |  |  |  |
|  | Purvez Grant, MD | Shweta Hande, BHMS, PGDCR | Pune | Ruby Hall Clinic,Grant Medical Foundation (10) |
|  |  | Poonam Sonawane, B.ScMicrobiology, ACCR | |  |
|  | Ranjan Kachru, MD | Abhishek Dubey | New Delhi | Fortis Healthcare Fl.t Lt. Rajan Dhall Hospital (4) |
|  |  | Kavita Rawat |  |  |
|  | Ajit Kumar VK, MD, DM |  | Trivandrum | Sree Chitra Tirunal Institute for Medical Sciences and Technology (3) |
|  | Sanjay Ganapathi, MD, DM |  |  |  |
|  | Jayakumar K, MS, M.Ch | Vineeth CP |  |  |
|  | Harikrishnan Sivadasanpillai, MD, DM | Manas Chacko, RN |  |  |
|  | Bijulal Sasidharan, MD, DM | Suresh Babu |  |  |
|  | Kapilamoorthy TR, MD |  |  |  |
|  | Johann Christopher, MD | Sowjanya Reddy | Hyderabad | CARE Hospital (3) |
|  | Praneeth Polamuri, MD | Manjula Rani |  |  |
|  | Upendra Kaul, MD | Priyadarshani Arambam | New Delhi | Batra Hospital and Medical Research Centre (BHMRC) (3) |
|  |  | Bebek Singh |  |  |
| United Kingdom (539) |  |  |  |  |
| Country Leaders |  |  |  |  |
| Roxy Senior, MBBS, MD, DM |  |  |  |  |
| Keith AA Fox, MBChB *(past)* |  |  |  |  |
| Country Coordinators |  |  |  |  |
| Grace M. Young , MSc, BSc (Hons) | |  |  |  |
| Kathryn Carruthers *(past)* |  |  |  |  |
|  | Roxy Senior, MBBS, MD, DM |  | Harrow | Northwick Park Hospital Harrow/ Royal Brompton Hospital London (202) |
|  | Ahmed Elghamaz, MB BCh |  |  |  |
|  | Sothinathan Gurunathan, MBChB |  |  |  |
|  | Nikolaos Karogiannis, MBBS | Grace M. Young , MSc, BSc (Hons) |  |  |
|  | Benoy N Shah, MD, MBBS, BSc (Hons) | Christopher Kinsey |  |  |
|  | Richard HJ Trimlett, MBBS, CCST | Raisa Kavalakkat, MSc, BSc, RN |  |  |
|  | Michael B Rubens, LRCP, MRCS, MBBS, DMRD | Jo Evans, RN |  |  |
|  | Edward D Nicol, MD, BMedSci, MBBS, DTM&H | Ikraam Hassan, RN |  |  |
|  | Tarun K Mittal, MD |  |  |  |
|  | Reinette Hampson, BSc (Hons), BA (Hons) | |  |  |
|  | Reto Andreas Gamma, MBBS | Sarah Williams, RN | Chelmsford | Broomfield Hospital (39) |
|  |  | Kim Holland, RN |  |  |
|  |  | Karen Swan, RN |  |  |
|  | Mark A de Belder, MD | Bev Atkinson, RN | Middlesbrough | The James Cook University Hospital, Middlesbrough (37) |
|  | Jeet Thambyrajah, MD |  |  |  |
|  | Thuraia Nageh, BSc(Hons) MBBS MD MRCP | Swapna Kunhunny, MRes Clin Res, BSc (N), RN | Westcliffe on Sea | Southend University Hospital (34) |
|  | John R Davies, MBBS, PhD |  |  |  |
|  | Steven J. Lindsay, MD | Craig Atkinson, RN | Bradford | Bradford Royal Infirmary (20) |
|  | John Kurian, MD | Carita Krannila, RN |  |  |
|  | Haqeel Jamil, MD | Manitha Vinod, RN |  |  |
|  | Osama Raheem, MD |  |  |  |
|  | Angela Hoye, MD | Lisa Chaytor | Cottingham | The University of Hull/Castle Hill Hospital (19) |
|  |  | Leanne Cox |  |  |
|  |  | Julie Morrow |  |  |
|  |  | Kay Rowe |  |  |
|  | Patrick Donnelly, MD | Stephanie Kelly, RN | Belfast | South Eastern Health and Social Care (17) |
|  | Bernardas Valecka, MD | Susan Regan, RN |  |  |
|  |  | Dawn Turnbull |  |  |
|  | Anoop Chauhan, MD | Catherine Fleming | Blackpool | Blackpool Teaching Hospitals (16) |
|  |  | Arijit Ghosh |  |  |
|  |  | Karen Gratrix |  |  |
|  |  | Stephen Preston |  |  |
|  | Craig Barr, MD | Anne Cartwright | Dudley | Russells Hall Hospital (15) |
|  | Khaled Alfakih, MBBS, MD | Abigail Knighton, BSc., PG Dip. | London | King's College NHS Foundation Hospital (14) |
|  | Jonathan Byrne, PhD | Katherine Martin, RGN, Dip. N, MSc |  |  |
|  | Ian Webb, PhD, MA |  |  |  |
|  | Peter Henriksen, PhD, MB ChB, BSc(Hons) | Laura Flint, RGN | Edinburgh | Royal Infirmary of Edinburgh (13) |
|  |  | James Harrison, BSc(Hons), PG dip |  |  |
|  | Peter OKane, MD | Nicki Lakeman | Bourneouth | Royal Bournemouth Hospital (13) |
|  |  | Anja Ljubez |  |  |
|  | Ramesh de Silva, MB ChB, MD |  | Bedford | Bedford Hospital NHS Trust (11) |
|  | Dwayne S. G. Conway, MD | Judith Wright | Wakefield | Pinderfields Hospital (11) |
|  |  | Donna Exley |  |  |
|  | Alexander A Sirker, MB BChir, PhD |  | London |  |
|  |  | Mervyn Andiapen, RN |  | University College London Hospitals NHS Foundation Trust |
|  |  | Amy J. Richards, BSc |  | BartsHealth NHS Trust |
|  |  |  |  |  |
|  |  |  |  | -11 |
|  | Stephen P Hoole, MD | Lisa Wong, MSc | Cambridge | Papworth Hospital (10) |
|  | Fraser N. Witherow, MD | Melanie J. Munro, RGN | Dorchester | Dorset County Hospital (8) |
|  | Nicola Johnston, MB, Bch BAO, MRCP, MD | | Belfast | Belfast Trust (7) |
|  | Mark Harbinson, MB, Bch BAO, MRCP, MD | Michelle McEvoy, RN |  |  |
|  | Simon Walsh, MB, Bch BAO, MD | Caroline Brown, RN |  |  |
|  | Hanna Douglas, MB, Bch BAO, MRCP, MD | |  |  |
|  | Matthew Luckie, MD | Thabitha Charles | Manchester | Central Manchester University Hospital (7) |
|  |  | Laurel Kolakaluri |  |  |
|  |  | Hannah Phillips |  |  |
|  | Jolanta Sobolewska, MD | Louise Morby, RN | Oldham | The Pennine Acute Hospitals NHS Trust (6) |
|  |  | Karen Hallett, RN |  |  |
|  |  | Carolyn Corbett, RN |  |  |
|  |  | Lynne Winstanley |  |  |
|  | Paramjit Jeetley, MD | Angelique Smit, RN | London | Royal Free London NHS Foundation Trust (6) |
|  | Niket Patel, MD |  |  |  |
|  | Tushar Kotecha, MBChB, Mpharm |  |  |  |
|  | Christopher Travill, MBBS, MD | Susan Gent, SRN RGN | Luton | Luton and Dunstable University Hospital NHS FT (5) |
|  | Iqbal Karimullah, MBBS | Nafisa Hussain, BSc |  |  |
|  | Mahmud Al-Bustami, MBBS |  |  |  |
|  | Denise Braganza, MD | Fiona Haines | Peterborough | Peterborough City Hospital (5) |
|  |  | Joanne Taaffe |  |  |
|  | Robert Henderson, MD | Jane Burton | Nottingham | Nottingham University Hospitals (4) |
|  | Kate Pointon, MBBS | Maria Colton |  |  |
|  | Surendra Naik, PhD | Rachel King |  |  |
|  | Thomas Mathew, MBBS, MD, DM |  |  |  |
|  |  | Ammani Brown, MSc BA RN | Clydebank | University of Glasgow (4) |
|  |  | Andrew Docherty, RN |  |  |
|  | Colin Berry, BSc MB ChB, PhD | Lisa McCloy, RN |  |  |
|  | Damien Collison, MB ChB | Kate Robb, RN |  |  |
|  | Giles Roditi, MB ChB | Craig Paterson, PhD |  |  |
|  |  | Wenda Crawford, RN |  |  |
|  |  | Joanne Kelly, RN |  |  |
|  |  | Lorraine McGregor, RN |  |  |
|  | Andrew J Moriarty, BSc MB PhD | Anne Mackin, RN, BSc | Craigavon | Cardiovascular Research Unit, Craigavon Area Hospital (2) |
|  | Jason D. Glover, MBBS | Janet P Knight, RN | Basingstoke | Hampshire Hospitals NHS Foundation Trust (2) |
|  | Jiwan Pradhan, MBBS |  |  |  |
|  | Ghada Mikhail, MD | Tuhina Bose | London | Imperial College Healthcare NHS Trust (1) |
|  | Darrel P. Francis, MD, MA |  |  |  |
| *Canada (447) |  |  |  |  |
| Country Leaders |  |  |  |  |
| Vladimir Dzavik, MD |  |  |  |  |
| Shaun Goodman, MD, MSc |  |  |  |  |
| Gilbert Gosselin, MD |  |  |  |  |
|  | Gilbert Gosselin, MD | Anna Proietti, RN | Montreal, QC | Montreal Heart Institute (90) |
|  |  | Myriam Brousseau, RN |  |  |
|  |  | Magalie Corfias, RN |  |  |
|  |  | Patricia Blaise |  |  |
|  |  | Luc Harvey |  |  |
|  | Ariel Diaz, MD |  | Trois-Rivieres, QC | Centre Hospitalier de Regional Trois-Rivieres (71) |
|  | Philippe Rheault, MD |  |  |  |
|  | Miguel Barrero, MD |  |  |  |
|  | Carl-Éric Gagné, MD | Patricia Alarie |  |  |
|  | Yanek Pépin-Dubois, MD | Linda Arcand |  |  |
|  | Ricardo Costa, MD | Isabelle Roy |  |  |
|  | Ying Tung Sia, MD | Estelle Montpetit |  |  |
|  | Catherine Lemay, MD |  |  |  |
|  | Alejandro Gisbert, MD |  |  |  |
|  | Pierre Gervais, MD |  |  |  |
|  | Alain Rheault, MD |  |  |  |
|  |  | Katia Drouin, RN | Terrebonne, QC | CISSSL - Hopital Pierre-Le Gardeur (42) |
|  | Denis Carl Phaneuf, MD | Christine Bergeron, RN |  |  |
|  | Gilbert Gosselin, MD | Christine Shelley |  |  |
|  |  | Christine Masson |  |  |
|  | Pallav Garg, MBBS, MSc | Sandy Carr, RN | London, ON | London Health Sciences Centre (35) |
|  |  | Catherine Bone, RN |  |  |
|  | Benjamin J.W. Chow, MD | Ermina Moga | Ottawa, ON | University of Ottawa Heart Institute (29) |
|  | Renee C. Hessian, MD | Janetta Kourzenkova |  |  |
|  | Rob S. Beanlands, MD | Olga Walter |  |  |
|  | Richard F. Davies, MD |  |  |  |
|  | Kevin R. Bainey, MD, MSc | Norma Hogg, RN | Edmonton, AB | University of Alberta (28) |
|  |  | Suzanne Welsh, RN |  |  |
|  | Asim N. Cheema, MD, PhD |  | Toronto, ON | St. Michael's Hospital (27) |
|  | Akshay Bagai, MD, MHS |  |  |  |
|  | Ron Wald, MDCM, MPH |  |  |  |
|  | Shaun Goodman, MD, MSc | Khrystyna Kushniriuk, HBSc, MD |  |  |
|  | John Joseph Graham, MRCP, MB ChB, BSc | Mohammed Hussain |  |  |
|  | Mark Peterson, MD, FRCSC, PhD | Olugbenga Bello |  |  |
|  | Chi-Ming Chow, MD, CM, MSc |  |  |  |
|  | Beth Abramson, MD, MSc |  |  |  |
|  | Asim Nazir Cheema, MD | Ishba Syed, MBBS | Mississauga, ON | Dixie Medical Group (24) |
|  | Mohammad Tariq Vakani, MD | Mohammed Hussain, BSc(H) |  |  |
|  |  | Khrystyna Kushniriuk, MBBS |  |  |
|  | James Cha, MD | Judy Otis, CRC | Oshawa, ON | Dr. James Cha (21) |
|  |  | Rebecca Otis, CRC |  |  |
|  | Andrew G Howarth, MD, PhD | Michelle M Seib, RN | Calgary, AB | University of Calgary (15) |
|  |  | Sandra M Rivest, RN |  |  |
|  |  | Rosa Sandonato, BSCN |  |  |
|  | Graham Wong, MD | Jackie Chow | Vancouver, BC | Vancouver General Hospital (15) |
|  |  | Andrew Starovoytov |  |  |
|  |  | Naomi Uchida |  |  |
|  |  | Ngaire Meadows |  |  |
|  | Amar Uxa, MD | Nadia Asif | Toronto, ON | University Health Network (14) |
|  |  | Suzana Tavares |  |  |
|  | Paul Galiwango, MD | Bev Bozek, RN, CCRC | Scarborough, ON | Scarborough Cardiology Research (9) |
|  | Saleem Kassam, MD | Maria Shier |  |  |
|  | Ashok Mukherjee, MD | Lori-Ann Larmand |  |  |
|  | A. Joseph Ricci, MD | Amir Janmohamed |  |  |
|  |  | Brenda Hart |  |  |
|  | Andy Lam, MD | Jane Marucci | East Grimsby, ON | West Lincoln Memorial Hospital (8) |
|  |  | Sharon Tai |  |  |
|  | Shamir Mehta, MD | Sonya Brons, RN | Hamilton, ON | Hamilton General Hospital (7) |
|  |  | Chris Beck, RN |  |  |
|  |  | Glenda Wong, RN |  |  |
|  |  | Krystal Etherington |  |  |
|  |  | Thippeekaa Arumairajah |  |  |
|  | Jacob Udell, MD | Maria Aprile | Toronto, ON | Women's College Hospital (7) |
|  |  | Sara Karlsson |  |  |
|  |  | Susan Webber |  |  |
|  | Philippe Généreux, MD | Chantale Mercure | Montréal, QC | Centre Intégré Universitaire de Santé et de Services Sociaux du Montréal (2) |
|  | Adnan Hameed, MD | Nancy Aedy | St. Catharines, ON | Saint Catharines General Hospital (2) |
|  |  |  |  |  |
|  | Ledjalem Daba, MD | Fran Farquharson | Vaughan, ON | Northwest GTA Cardiovascular and Heart Rhythm Program (1) |
|  |  | Anam Siddiqui |  |  |
| Brazil (399) |  |  |  |  |
| Country Leaders |  |  |  |  |
| Antonio Carlos Carvalho, MD, PhD | |  |  |  |
| Renato D. Lopes, MD, PhD |  |  |  |  |
|  | Whady Hueb, MD | Myrthes Emy Takiuti, RN | Sao Paulo | Heart Institute (InCor) University of São Paulo (127) |
|  | Paulo Cury Rezende, MD |  |  |  |
|  | Expedito Eustáquio Ribeiro Silva, MD |  |  |  |
|  | Alexandre Ciappina Hueb, MD |  |  |  |
|  | Paola Emanuela Poggio Smanio, MD, PhD | Leonardo Pizzol Caetano, PhD | São Paulo | Instituto Dante Pazzanese de Cardiologia (98) |
|  | Alexandre Schaan de Quadros, MD |  | Porto Alegre | Instituto de Cardiologia de Porto Alegre (41) |
|  | Renato Abdala Karam Kalil, MD | Aline Peixoto Deiro |  |  |
|  | José Luiz da Costa Vieira, MD | Alice Manica Muller |  |  |
|  | Gabriel Grossmann , MD | Maria Antonieta Pereira de Moraes |  |  |
|  | Pedro Píccaro de Oliveira, MD | Bruna Maria Ascoli |  |  |
|  | Leonardo Bridi, MD | Sílvia Zottis Poletti |  |  |
|  | Simone Savaris, MD |  |  |  |
|  | João V Vitola, MD, PhD |  | Curitiba | Quanta Diagnostico & Terapia (33) |
|  | Rodrigo J Cerci, MD, Msc | Sandra S. Zier, BSc |  |  |
|  | Fabio R Farias, MD, Msc | Vilmar Veiga Jr, BSc |  |  |
|  | Miguel M Fernandes, MD, PhD |  |  |  |
|  | José Antonio Marin-Neto, MD, PhD |  | Ribeirao Preto | Hospital das Clinicas da Faculdade de Medicina de Ribeirão Preto da Universidade de São Paulo (31) |
|  | André Schmidt, MD, PhD |  |  |  |
|  | Moysés de Oliveira Lima Filho, MD, PhD | Diego Franca da Cunha |  |  |
|  | Ricardo Mendes Oliveira, MD |  |  |  |
|  | João Reynaldo Abbud Chierice, MD |  |  |  |
|  | Carísi A. Polanczyk, MD | Guilherme G Rucatti, PsyD | Porto Alegre | Hospital de Clínicas de Porto Alegre |
|  | Mariana V. Furtado, MD | Fernanda Igansi, BSc |  | -12 |
|  | Luis F. Smidt, MD | Mauren P Haeffner, BSc |  |  |
|  | Antonio Carlos Carvalho, MD | Viviane Almeida | Sao Paulo | Unifesp - Hospital Sao Paulo (9) |
|  | Gustavo Pucci, MD | Gabriela Sanchez de Souza |  |  |
|  | Flavio Lyra, MD |  |  |  |
|  | Alvaro Rabelo Alves Junior, MD | Mayana Almeida | Salvador | Fundacao Bahiana de Cardilogia (9) |
|  |  | Viviane dos Santos |  |  |
|  | Marianna D. A. Dracoulakis, MD, PhD | Natalia S Oliveira, RN | Salvador | Hospital da Bahia (8) |
|  | Rodolfo G. S. D Lima, MD |  |  |  |
|  | Estevao Figueiredo, MD | Bruna Edilena Paulino Azevedo | Belo Horizonte | Hospital Lifecenter (8) |
|  | Paulo Ricardo Caramori, MD | Marco Bizzaro Santos | Porto Alegre | Hospital Sao Lucas da Pontificia Universidade Catolica do Rio Grande do Sol (7) |
|  |  | Amanda Germann |  |  |
|  |  | Vitor Gomes |  |  |
|  |  | Rosa Homem |  |  |
|  |  | Ellen Magedanz |  |  |
|  | Rogerio Tumelero, MD | Rosane Laimer | Fundo | Hospital Sao Vicente de Paulo (5) |
|  |  | Alexandre Tognon |  |  |
|  | Frederico Dall’Orto, MD |  | Pocos de Caldas | Hospital Maternidade e Pronto Socorro Santa Lucia (4) |
|  | Claudio T. Mesquita, MD | Roberta P Santos, RN | Botafogo | Hospital Pró-Cardíaco (3) |
|  | Alexandre S. Colafranseschi, MD |  |  |  |
|  |  |  |  |  |
|  | Amarino C. Oliveira Jr., MD |  |  |  |
|  | Luiz A. Carvalho, MD |  |  |  |
|  | Isabella C. Palazzo, MD |  |  |  |
|  | Andre S. Sousa, MD |  |  |  |
|  | Expedito Eustáquio Ribeiro da Silva, MD, PhD | | Sao Paulo | Hospital TotalCor (2) |
|  | Pedro Gabriel Melo de Barros e Silva, MD, PhD | Mariana Yumi Okada, RN |  |  |
|  | Luciana de Pádua Silva Baptista, MD, PhD | Ana Paula Batista, RN |  |  |
|  | Marcelo Jamus Rodrigues, MD | Aline Nogueira Rabaça, BS |  |  |
|  | Marcos Valério Coimbra de Resende, MD, PhD | |  |  |
|  | Jose Francisco Saraiva, MD | Larissa Miranda Trama | Sao Paulo | Hospital Celso Pierro (1) |
|  |  | Talita Silva |  |  |
|  |  | Camila Thais de Souza Ormundo |  |  |
|  |  | Carla Vicente |  |  |
|  | Costantino Costantini, MD, PhD | Caroline Pinheiro | Curitiba | Hospital Cardiologico Costantini (1) |
|  |  | Daniele Komar |  |  |
| Poland (333) |  |  |  |  |
| Country Leaders |  |  |  |  |
| Witold Ruzyllo, MD |  |  |  |  |
| Hanna Szwed, MD, PhD |  |  |  |  |
| Country Coordinator |  |  |  |  |
| Radoslaw Pracon, MD, PhD |  |  |  |  |
|  | Marcin Demkow, MD, PhD |  | Warsaw | Coronary and Structural Heart Diseases Department, Institute of Cardiology (127) |
|  | Radoslaw Pracon, MD, PhD |  |  |  |
|  | Cezary Kepka, MD PhD |  |  |  |
|  | Anna Teresinska, MD PhD | Olga Walesiak |  |  |
|  | Karolina Kryczka, MD PhD | Katarzyna Malinowska |  |  |
|  | Jan Henzel, MD PhD |  |  |  |
|  | Mateusz Solecki, MD PhD |  |  |  |
|  | Edyta Kaczmarska, MD PhD |  |  |  |
|  | Tomasz Mazurek, MD, PhD | Jakub Maksym, MD | Warszawa | Medical University of Warsaw (48) |
|  |  | Karolina Wojtera, MD |  |  |
|  |  | Anna Fojt, MD |  |  |
|  |  | Ewa Szczerba, MD |  |  |
|  | Jaroslaw Drozdz, PhD |  | Lodz | Cardiology Clinic, Medical University in Lodz (43) |
|  | Bartosz Czarniak, MD |  |  |  |
|  | Malgorzata Frach (formerly Stasiak), MD |  |  |  |
|  | Konrad Szymczyk, MD |  |  |  |
|  | Iwona Niedzwiecka, MD |  |  |  |
|  | Sebastian Sobczak, MD |  |  |  |
|  | Tomasz Ciurus, MD |  |  |  |
|  | Piotr Jakubowski, MD |  |  |  |
|  | Magdalena Misztal-Teodorczyk, MD |  |  |  |
|  | Dawid Teodorczyk, MD | Marta Swiderek, MA |  |  |
|  | Aleksandra Fratczak, MD | Ewelina Wojtala, MA |  |  |
|  | Marcin Szkopiak, MD |  |  |  |
|  | Patrycja Lebioda, MD |  |  |  |
|  | Michal Wlodarczyk, MD |  |  |  |
|  | Anna Plachcinska, MD |  |  |  |
|  | Jacek Kusmierek, MD |  |  |  |
|  | Magdalena Miller, MD |  |  |  |
|  | Halina Marciniak, MD |  |  |  |
|  | Karolina Wojtczak-Soska, MD |  |  |  |
|  | Katarzyna Łuczak, MD |  |  |  |
|  | Tomasz Tarchalski, MD |  |  |  |
|  | Anna Cichocka-Radwan, MD |  |  |  |
|  | Hanna Szwed, MD, PhD | Jaroslaw Karwowski, MD | Warsaw | National Institute of Cardiology, Warsaw (35) |
|  | Grazyna Anna Szulczyk, MD |  |  |  |
|  | Adam Witkowski, MD, PhD |  | Warsaw | Department of Interventional Cardiology & Angiology, Institute of Cardiology (20) |
|  | Krzysztof Kukuła, MD, PhD |  |  |  |
|  | Małgorzta Celińska-Spodar, MD |  |  |  |
|  | Joanna Zalewska, MD |  |  |  |
|  | Grzegorz Gajos, MD, PhD |  | Krakow | Department of Coronary Disease, John Paul II Hospital, Jagiellonian University Medical College  (16) |
|  | Krzysztof Bury, MD, PhD |  |  |  |
|  | Piotr Pruszczyk, MD, PhD | Andrzej Łabyk, MD | Warszawa | Department of Internal Medicine and Cardiology, Infant Jesus Teaching Hospital, Medical University of Warsaw (15) |
|  | Marek Roik, MD, PhD | Agnieszka Szramowska, MD |  |  |
|  |  | Olga Zdończyk, MD |  |  |
|  | Krystyna Łoboz-Grudzień, MD, PhD | Joanna Jaroch, MD, PhD | Wrocław | T.Marciniak Hospital (11) |
|  | Leszek Sokalski, MD, PhD |  |  |  |
|  | Barbara Brzezińska, MD, PhD |  |  |  |
|  | Maciej Lesiak, Professor, MD |  | Poznan | Szpital Kliniczny Przemienienia Pańskiego (10) |
|  | Magdalena Łanocha, MD |  |  |  |
|  | Krzysztof W. Reczuch, MD | Adam Kolodziej, MD | Wroclaw | Military Hospital / Medical University (4) |
|  | Zbigniew Kalarus, MD |  | Zabrze | Medical University of Silesia, School of Medicine with the Division of Dentistry, Department of Cardiology, Congenital Heart Diseases and Electrotherapy, Silesian Center for Heart Diseases (3) |
|  | Andrzej Swiatkowski, MD |  |  |  |
|  | Mariola Szulik, MD |  |  |  |
|  | Wlodzimierz J. Musial, MD | Marta Marcinkiewicz-Siemion, MD | Bialystok | University Hospital in Bialystok (1) |
| Russia (303) |  |  |  |  |
| Country Coordinator |  |  |  |  |
| Olga Bockeria, MD, PhD |  |  |  |  |
|  | Leo Bockeria, MD, PhD | Olga Bockeria, MD, PhD | Moscow | National Medical Research Center for Cardiovascuar Surgery (113) |
|  | Karen Petrosyan, MD, PhD | Zalina Kudzoeva, MD |  |  |
|  | Tatiana Trifonova, MD | Nodira Aripova, MD |  |  |
|  | Alexander M. Chernyavskiy, MD, PhD | Ivan A. Naryshkin, MD | Novosibirsk | E.Meshalkin National Medical Research Center of the Ministry of Health of the Russian Federation (101) |
|  | Evgeniy I. Kretov, MD | Alena Kuleshova, MD |  |  |
|  | Igor O. Grazhdankin, MD | Dastan Malaev, MD |  |  |
|  | Leonid L. Bershtein, MD, PhD |  | Saint Petersburg | North-Western State Medical University (50) |
|  | Sergey A. Sayganov, MD, PhD | Irina Subbotina |  |  |
|  | Anastasia M. Kuzmina-Krutetskaya, MD | Victoria Gumerova |  |  |
|  | Elizaveta V. Zbyshevskaya, MD, PhD |  |  |  |
|  | Nana O. Katamadze, MD, PhD |  |  |  |
|  | Elena A. Demchenko, MD, PhD | Olga B. Nikolaeva, MD | Saint Petersburg | Federal Almazov North-West Medical Research Centre (39) |
|  | Pavel S. Kozlov, MD |  |  |  |
|  | Vikentiy Y. Kozulin, MD |  |  |  |
|  | Ekaterina I. Lubinskaya, MD |  |  |  |
| *Spain (286) |  |  |  |  |
| Country Leader |  |  |  |  |
| Jose Luis Lopez-Sendon, MD, PhD | |  |  |  |
| Country Coordinator |  |  |  |  |
| Almudena Castro, MD |  |  |  |  |
|  | Jose Lopez-Sendon, MD, PhD | Virginia Fernández-Figares, Pharm | Madrid | Hospital La Paz. IdiPaz (118) |
|  | Almudena Castro, MD |  |  |  |
|  | Elena Refoyo Salicio, MD |  |  |  |
|  | Gabriela Guzman, MD |  |  |  |
|  | Gabriel Galeote, MD |  |  |  |
|  | Silvia Valbuena, MD |  |  |  |
|  | Jesús Peteiro, MD, PhD |  | A Coruna | Complexo Hospitalario Universitario A Coruña (CHUAC) Sergas, Department of Cardiology. INIBIC A Coruña. CIBER-CV. Universidad de A Coruña, Spain (112) |
|  | María Dolores Martínez-Ruíz, MD |  |  |  |
|  | Ruth Pérez-Fernández, MD | Moisés Blanco-Calvo, PhD |  |  |
|  | José J Cuenca-Castillo, MD | Encarnación Alonso-Álvarez, BSc |  |  |
|  | Xacobe Flores-Ríos, MD | Paula García-González, BSc |  |  |
|  | Óscar Prada-Delgado, MD |  |  |  |
|  | Gonzalo Barge-Caballero, MD |  |  |  |
|  | Jose Ramon Gonzalez Juanatey, MD, PhD | Jose Seijas Amigo, Pharm | Santiago de Compostela | Hospital Clinico Universitario de Santiago (17) |
|  | Miguel Souto Bayarri, MD, PhD |  |  |  |
|  | Virginia Pubull Nuñez, MD |  |  |  |
|  | Raymundo Ocaranza Sanchez, MD, PhD |  |  |  |
|  | Belen Cid Alvarez, MD |  |  |  |
|  | Carlos Peña Gil, MD, PhD |  |  |  |
|  | Amparo Martinez Monzonis, MD |  |  |  |
|  | Alessandro Sionis, MD | Ana Fernández Martínez, RN | Barcelona | Hospital de la Santa Creu i Sant Pau (11) |
|  | Montserrat Vila Perales, MD |  |  |  |
|  | Josep Maria Padró, MD |  |  |  |
|  | Antonio Serra Peñaranda, MD |  |  |  |
|  | Joan García Picart, MD |  |  |  |
|  | Antonino Ginel Iglesias, MD |  |  |  |
|  | Xavier Garcia-Moll Marimon, MD |  |  |  |
|  | Guillem Pons Lladó, MD |  |  |  |
|  | Francesc Carreras Costa, MD |  |  |  |
|  | Vicente Miro, MD | Begoña Igual, MD | Valencia | Hospital Universitario y Politecnico La Fe (10) |
|  | Jose L Diez, MD |  |  |  |
|  | Pilar Calvillo, MD |  |  |  |
|  | F. Marin Ortuño, MD, PhD |  | Murcia | HUVA, Hospital Clínico Universitario Virgen De La Arrixaca (8) |
|  | M. Valdés Chávarri, MD, PhD | M. Quintana Giner, MD |  |  |
|  | A. Tello Montolliu, MD, PhD | A.I. Romero Aniorte, MD |  |  |
|  | E. Pinar Bermudez, MD, PhD | JM. Rivera Caravaca, MD |  |  |
|  | G. De La Morena, MD, PhD |  |  |  |
|  | Montserrat Gracida Blancas, MD | Olga Cañavate | Barcelona | Hospital De Bellvitge (4) |
|  |  | Sonia Guerrero |  |  |
|  |  | Silvia Riera |  |  |
|  | Jose Enrique Castillo Luena, MD | Jose Enrique Castillo Luena | Zaragoza | Hospital Universitario Miguel Servet (4) |
|  |  | Maria Lasala |  |  |
|  | Francisco Fernandez-Aviles, MD | Maria Lorenzo | Madrid | Hospital General Universitario Gregorio Maranon (2) |
|  |  | Olga Sobrino |  |  |
|  |  | Alexandra Vazquez |  |  |
| China (246) |  |  |  |  |
| Country Leader |  |  |  |  |
| Lixin Jiang, MD, PhD |  |  |  |  |
|  | Jiyan Chen, MD | Haojian Dong | Guangzhou | Guangdong General Hospital (102) |
|  |  | Peiyu He |  |  |
|  |  | Chunli Xia |  |  |
|  |  | Junqing Yang |  |  |
|  |  | Qi Zhong |  |  |
|  | Yongjian Wu, MD, PhD | Yanmeng Tian, MD | Beijing | Chinese Academy of Medical Sciences, Fuwai Hospital (17) |
|  |  | Dongze Li | Urumqi | First Affiliated Hospital of Xinjiang Medical University (15) |
|  | Yitong Ma, MD | Xiaomei Li |  |  |
|  | Yining Yang, MD | Xiang Ma |  |  |
|  |  | Zixiang Yu |  |  |
|  |  | Qian Zhao |  |  |
|  | Zheng Ji, MD | Chunguang Li | Tangshan | Tangshan Gongren Hospital (15) |
|  |  | Lei Zhang |  |  |
|  |  | Yu Zhao |  |  |
|  |  | Bolin Zhu |  |  |
|  | Xinchun Yang, MD | Mulei Chen | Beijing | Beijing Chao-yang Hospital, Capital Medical University (12) |
|  |  | Hongjie Chi |  |  |
|  |  | Yang Wang |  |  |
|  |  | Jing Zhang |  |  |
|  | Wenhua Lin, MD | Rui Jing | Tianjing | TEDA International Cardiovascular Hospital (12) |
|  |  | Jingjing Liu |  |  |
|  | Hesong Zeng, MD | Qiang Zhou, MD | Wuhan | Tongji Medical College (11) |
|  |  | Chang Xu, MD |  |  |
|  |  | Zhuxi Li, MD |  |  |
|  |  | Junhua Li, MD |  |  |
|  |  | Luyang Xiong, MD |  |  |
|  | Xin Fu, MD | Dan Gao | Zhengzhou | The First Affiliated Hospital of Zhengzhou University (11) |
|  |  | Dengke Jiang |  |  |
|  |  | Ran Leng |  |  |
|  |  | Xutong Wang |  |  |
|  |  | Qianqian Yuan |  |  |
|  |  | Lili Zhang |  |  |
|  | Bin Yang, MD | Ziliang Bai | Taiyuan | Shanxi Cardiovascular Hospital (10) |
|  |  | Jianhua Li |  |  |
|  |  | Jie Qi |  |  |
|  |  | Fei Wang |  |  |
|  |  | Haitao Wang |  |  |
|  |  | Bin Yang |  |  |
|  |  | Zhou Yue |  |  |
|  |  | Zhulin Zhang |  |  |
|  | Songtao Wang, MD | Yumei Dong | Qingdao | Qingdao Fuwai Hospital (8) |
|  |  | Jiajia Mao |  |  |
|  |  | Bin Zhang |  |  |
|  | Gong Cheng, MD | Xiuhong Li | Xian | Shanxi Provincial People’s Hospital (6) |
|  |  | Xiaowei Yao |  |  |
|  |  | Nier Zhong |  |  |
|  |  | Ning Zhou |  |  |
|  | Yulan Zhao, MD | Yaping Huang, MS | Zhengzhou | The Second Affiliated Hospital of Zhengzhou University (6) |
|  |  | Panpan Zhou, MS |  |  |
|  | Xuehua Fang, MD | Wei Su | Beijing | Liangxiang Hospital, Beijing Fangshan District (6) |
|  | Qiutang Zeng, MD | Yu Kunwu | Wuhan | Wuhan Union Hospital, Tongji Medical College, Huazhong Science and Tech University (3) |
|  |  | Yudong Peng |  |  |
|  |  | Xin Su |  |  |
|  | Xi Su, MD | Chen Wang | Wuhan | Wuhan Asia Heart Hospital (3) |
|  |  | Yunhai Zhao |  |  |
|  | Qingxian Li, MD | Yaming Geng | Jining | Affiliated Hospital of Jining Medical University (3) |
|  |  | Yanfu Wang |  |  |
|  | Shao-ping Nie, MD, PhD | Jing-yao Fan, MD | Beijing | Beijing Anzhen Hospital (2) |
|  |  | Si-ting Feng, MD,PhD |  |  |
|  |  | Xiao Wang, MD,PhD |  |  |
|  |  | Yan Yan, MD,PhD |  |  |
|  |  | Hui-min Zhang, MD,PhD |  |  |
|  | Qin Yu, MD | Lingping Chi | Dalian | Affiliated Zhongshan Hospital of Dalian University (2) |
|  |  | Fang Liu |  |  |
|  | Jian'an Wang, MD | Han Chen | Hangzhou | The Second Affiliated Hospital Zhejiang University School of Medicine (1) |
|  |  | Jun Jiang |  |  |
|  |  | Huajun Li |  |  |
|  |  | Jian'an Wang |  |  |
|  |  | Yechen Han, MM | Beijing | Peking Union Medical College Hospital (1) |
|  |  | Lihong Xu, RN |  |  |
|  | Shuyang Zhang, MD, PhD | Zhenyu Liu |  |  |
|  | Zhenyu Liu, MD | Gang Chen |  |  |
|  |  | Rongrong Hu |  |  |
| *Italy (139) |  |  |  |  |
| Country Leader |  |  |  |  |
| Aldo P. Maggioni, MD |  |  |  |  |
|  |  |  |  |  |
|  | Gian Piero Perna, MD | Francesca Pietrucci, PhD | Ancona | Cardiology and CCU - Ospedali Riuniti Ancona (54) |
|  | Marco Marini, MD |  |  |  |
|  | Gabriele Gabrielli, MD |  |  |  |
|  | Stefano Provasoli, MD | Anna Di Donato | Varese | Ospedale di Circolo e Fondazione Macchi (23) |
|  | Edoardo Verna, MD |  |  |  |
|  | Lorenzo Monti, MD |  | Rozzano | Humanitas Research Hospital, Rozzano (MI) (17) |
|  | Barbara Nardi, MD |  |  |  |
|  | Antonio Di Chiara, MD | Francesca Pezzetta, MD | Tolmezzo | Azienda Servizi Sanitaria n.3 Alto Friuli-Collinare-Medio Friuli (9) |
|  | Andrea Mortara, MD | Valentina Casali, MD | Monza | Policlinico di Monza, Monza MB (8) |
|  | Marcello Galvani, MD | Chiara Attanasio | Forli | Ospedale “G.B. Morgagni – L. Pierantoni” Forli (AUSL della Romagna) (8) |
|  | Filippo Ottani, MD |  |  |  |
|  | Marco Sicuro, MD | Gianpiero Leone, MD | Aosta | Ospedale Regionale Umberto Parini (5) |
|  |  | Francesco Pisano, MD |  |  |
|  |  | Cristina Bare, BSc |  |  |
|  | Paolo Calabro, MD | Fabio Fimiani | Napoli | AORN Dei Colli "V. Monaldi" UOC Cardiologia Università della Campania "L.Vanvitelli" (4) |
|  | Tiziana Formisano, MD |  |  |  |
|  | Giuseppe Tarantini, MD | Alberto Barioli, MD | Padua | University of Padua- Cardiology Clinic (3) |
|  | Umberto Cucchini, MD | Federica Ramani |  |  |
|  | Anto Luigi Andres, MD |  |  |  |
|  | Emanuela Racca, MD | Fabrizio Rolfo, MD | Cuneo | Azienda Ospedaliera S. Croce e Carle (3) |
|  |  | Cecilia Goletto |  |  |
|  | Carlo Briguori, MD | Francesca De Micco | Naples | Clinica Mediterranea (2) |
|  | Roberto Amati, MD | Stefano Di Marco, MD | Pescia | UO Cardiologia Ospedale SS Cosma e Damiano (2) |
|  | William Vergoni, MD | Martina Tricoli |  |  |
|  | Aldo Russo, MD | Massimo Villella, MD | San Giovanni Rotondo | IRCCS "Casa Sollievo della Sofferenza" (1) |
|  | Raffaele Fanelli, MD |  |  |  |
| *Singapore (61) |  |  |  |  |
| Country Leader |  |  |  |  |
| Harvey Douglas White, MD |  |  |  |  |
| Country Coordinator |  |  |  |  |
| Caroline Alsweiler |  |  |  |  |
|  | Kian-Keong Poh, MD |  | Singapore | National University Heart Center Singapore (33) |
|  | Ping Chai, MD |  |  |  |
|  | Titus Lau, MD |  |  |  |
|  | Joshua P. Loh, MD |  |  |  |
|  | Edgar L. Tay, MD |  |  |  |
|  | Kristine Teoh, MD | Sik-Yin V Tan, BSc |  |  |
|  | Lynette L. Teo, MD | Winnie C Sia, BSc |  |  |
|  | Ching-Ching Ong, MD | Audrey W Leong, BSc |  |  |
|  | Raymond C. Wong, MD |  |  |  |
|  | Poay-Huan Loh, MD |  |  |  |
|  | Theodoros Kofidis, MD |  |  |  |
|  | Wan Xian Chan, MD |  |  |  |
|  | Koo Hui Chan, MD |  |  |  |
|  | David Foo, MBBS | Li Hai Yan, RN | Singapore | Tan Tock Seng Hospital (22) |
|  | Jason Loh Kwok Kong, MD |  |  |  |
|  | Ching Min Er, MD |  |  |  |
|  | Fahim Haider Jafary, MD |  |  |  |
|  | Terrance Chua, MD | Nasrul Ismail | Singapore | National Heart Centre Singapore (6) |
|  |  | Min Tun Kyaw |  |  |
|  |  | Deborah Yip |  |  |
| Germany (54) |  |  |  |  |
| Country Leader |  |  |  |  |
| Rolf Doerr, MD |  |  |  |  |
|  | Rolf Doerr, MD |  | Dresden | Praxisklinik Herz und Gefaesse (29) |
|  | Juergen Stumpf, MD | Dorit Grahl |  |  |
|  | Klaus Matschke, MD, PhD | Franziska Guenther |  |  |
|  | Gregor Simonis, MD, PhD | Kerstin Bonin |  |  |
|  | Clemens T. Kadalie, MD |  |  |  |
|  | Udo Sechtem, MD | Ina Wenzelburger | Stuttgart | Robert-Bosch-Krankenhaus (22) |
|  | Peter Ong, MD | Susanne Gruensfelder, RN |  |  |
|  | P. Christian Schulze, MD, PhD |  | Jena | University Hospital Jena (2) |
|  | Bjoern Goebel, MD |  |  |  |
|  | Karsten Lenk, MD |  |  |  |
|  | Georg Nickenig, MD | Jan-Malte Sinning, MD | Bonn | Universitatsklinikum Bonn (1) |
|  |  | Marcel Weber, MD |  |  |
|  |  | Nikos Werner, MD |  |  |
| Austria (50) |  |  |  |  |
| Country Leaders |  |  |  |  |
| Irene Marthe Lang, MD |  |  |  |  |
| Kurt Huber, MD |  |  |  |  |
|  | Herwig Schuchlenz, MD | Gudrun Steinmaurer | Graz | LKH Graz West Austria (35) |
|  | Stefan Weikl, MD |  |  |  |
|  | Irene Marthe Lang, MD | Max-Paul Winter, MD | Vienna | Medical University of Vienna, Department of Cardiology (8) |
|  |  | Tijana, Andric, MD | Vienna | Wilhelminen Hospital Vienna (7) |
|  | Kurt Huber, MD | Maximilian, Tscharre, MD |  |  |
|  | Gabriele, Jakl-Kotauschek, MD | Claudia, Wegmayr, MSc |  |  |
|  |  | Bernhard, Jäger, MD |  |  |
|  |  | Florian, Egger, MD |  |  |
| Hungary (49) |  |  |  |  |
| Country Leader |  |  |  |  |
| Matyas Keltai, MD, PhD, DSc |  |  |  |  |
|  | Andras Vertes, MD | Judit Sebo, MD | Budapest | Eszszk- Szent Istvan Hospital (20) |
|  |  | Zoltan Davidovits, MD |  |  |
|  |  | Laszlone Matics |  |  |
|  | Albert Varga, MD, PhD | Gergely Ágoston, MD | Szeged | University of Szeged (12) |
|  | Geza Fontos, MD | Gabor Dekany, MD | Budapest | George Gottsegen National Institute of Cardiology (9) |
|  | Bela Merkely, MD, PhD, DSc | Andrea Bartykowszki, MD | Budapest | Heart and Vascular Center, Semmelweis University (8) |
|  |  | Pal Maurovich-Horvat, MD, PhD, MPH | |  |
|  | Gabor Kerecsen, MD | Agnes Jakal | Budapest | Military Hospital, Budapest (1) |
| Serbia (47) |  |  |  |  |
|  | Sasa Hinic, MD, BSc | Jelena Djokic, MD | Belgrade | University Hospital Center Bezanijska Kosa (13) |
|  | Marija Zdravkovic, MD, PhD |  |  |  |
|  | Vladan Mudrenovic, MD |  |  |  |
|  | Bogdan Crnokrak, MD |  |  |  |
|  | Branko D. Beleslin, MD, PhD |  | Belgrade | Faculty of Medicine, University of Belgrade; Cardiology Clinic, Clinical Center of Serbia (10) |
|  | Nikola N. Boskovic, MD | Ana D. Djordjevic-Dikic, MD, PhD |  |  |
|  | Marija T. Petrovic, MD | Vojislav L. Giga, MD, PhD |  |  |
|  | Milan R. Dobric, MD | Jelena J. Stepanovic, MD, PhD |  |  |
|  | Zeljko Z. Markovic, MD, PhD |  |  |  |
|  | Ana S. Mladenovic, MD, PhD |  |  |  |
|  | Nada Cemerlic-Adjic, MD | Lazar Velicki, MD | Sremska Kamenica | Institute of Cardiovascular Diseases Vojvodina, Sremska Kamenica, Serbia and Faculty of Medicine, University of Novi Sad (9) |
|  |  | Ljiljana Pupic |  |  |
|  | Goran Davidović, MD, PhD | Stefan M. Simović, MD | Kragujevac | Clinical Center Kragujevac (7) |
|  | Rada Vučić, MD |  |  |  |
|  | Milica Nikola Dekleva, MD PhD | Miroslav Stevo Martinovic, MD | Belgrade | University Clinical Hospital Zvezdara (6) |
|  |  | Gordana Stevanovic |  |  |
|  | Goran Stankovic, MD | Milan Dobric | Belgrade | Clinical Center of Serbia (1) |
|  | Svetlana Apostolovic, MD | Sonja Salinger Martinovic | Nis | Clinic for Cardiovascular Diseases, Clinical Center Nis (1) |
|  |  | Dragana Stanojevic |  |  |
| Mexico (46) |  |  |  |  |
|  | Jorge Escobedo, MD | Ramon de Jesús-Pérez, RN | Benito Juarez | Instituto Mexicano del Seguro Social (35) |
|  | Rubén Baleón-Espinosa, MD |  |  |  |
|  | Arturo S Campos-Santaolalla, MD |  |  |  |
|  | Elihú Durán-Cortés, MD |  |  |  |
|  | José M Flores-Palacios, MD |  |  |  |
|  | Andrés García-Rincón, MD |  |  |  |
|  | Moisés Jiménez-Santos, MD |  |  |  |
|  | Joaquín V Peñafiel, MD |  |  |  |
|  | José A Ortega-Ramírez, MD |  |  |  |
|  | Aquiles Valdespino-Estrada, MD |  |  |  |
|  | Erick Alexánderson Rosas, MD | María Fernanda Canales Brassetti, MD | Mexico City | Instituto Nacional de Cardiología "Ignacio Chávez" (11) |
|  |  | Diego Adrián Vences Anaya, MD |  |  |
|  |  | María Pérez García |  |  |
|  |  | Isabel Estela Carvajal Juarez , MD |  |  |
|  |  | Magdalena Madero Rovalo, MC |  |  |
|  |  | Erick Donato Morales Rodríguez, MD | |  |
| Australia (45) |  |  |  |  |
| Country Leaders |  |  |  |  |
| Joseph B. Selvanayagam, MBBS (Hons), DPhil | |  |  |  |
| Jamie Rankin, MBBS *(past)* |  |  |  |  |
| Country Coordinator |  |  |  |  |
| Deirdre Murphy |  |  |  |  |
|  | Joseph B. Selvanayagam, MBBS (Hons), DPhil | Sau Lee, PhD | Adelaide | Flinders Medical Centre (30) |
|  | Majo X. Joseph, MBBS | Prince Thomas, RN |  |  |
|  | Suku T. Thambar, MBBS | Melissa D Chaplin, RN | New Lambton Heights | John Hunter Hospital (8) |
|  |  | Stephanie C Boer, B Biotechnology (Honours) | |  |
|  | John F. Beltrame, MD | Jeanette K. Stansborough, RN | Woodville South | The Queen Elizabeth Hospital (5) |
|  |  | Marilyn Black, RN |  |  |
|  | Graham S. Hillis, PhD | Michelle M. Bonner, B. Nursing | Perth | Royal Perth Hospital (2) |
|  |  | Kim F. Ireland, RN |  |  |
|  |  | Clare Venn-Edmonds, RN |  |  |
| France (42) |  |  |  |  |
| Country Leader |  |  |  |  |
| Philippe-Gabriel Steg, MD |  |  |  |  |
| Country Coordinators |  |  |  |  |
| Helene Abergel |  |  |  |  |
| Jean-Michel Juliard |  |  |  |  |
|  |  | Corine Thobois, RN | Chartres | C.H. Louis Pasteur (21) |
|  | Christophe Thuaire, MD | Emilie Tachot, RN |  |  |
|  | Téodora Dutoiu, MD | Christophe Laure, RN |  |  |
|  |  | Christel Vassaliere, RN |  |  |
|  | Philippe Gabriel Steg, MD | Helene Abergel, MSc | Paris | Bichat Hospital (9) |
|  | Jean-Michel Juliard, MD | Axelle Fuentes, MSc |  |  |
|  | Michel S. Slama, MD | Ludivine Eliahou, MD | Clamart Cedex | Antoine-Beclere Hospital (5) |
|  | Rami El Mahmoud, MD | Olivier Dubourg, MD | Boulogne | Ambroise Pare Hospital (2) |
|  |  | Pierre Michaud, MD |  |  |
|  | Eric Nicollet, MD | Sarah Hadjih | Corbeil-Essonnes Cedex | Centre Hospitalier Sud Francilien (2) |
|  | Pascal Goube, MD | Patricia Brito |  |  |
|  | Gilles Barone-Rochette, MD | Gilles Barone-Rochette | Grenoble | Grenoble University Hospital (2) |
|  | Alain Furber, MD | Charles Cornet, MD, PhD | Angers Cedex 9 | Centre Hospitalier Universitaire d'Angers (1) |
|  | Loïc Bière, MD | Jeremy Rautureau, MD, PhD |  |  |
| Lithuania (39) |  |  |  |  |
|  |  | Agne Juceviciene, MD | Vilnius | Vilnius University Hospital Santariskes Clinic (39) |
|  |  | Irma Kalibataite-Rutkauskiene, MD |  |  |
|  |  | Laura Keinaite |  |  |
|  | Aleksandras Laucevicius, MD | Monika Laukyte |  |  |
|  | Jelena Celutkiene, MD | Gelmina Mikolaitiene |  |  |
|  |  | Akvile Smigelskaite, MD |  |  |
|  |  | Ilona Tamasauskiene, MD |  |  |
|  |  | Agne Urboniene, MD |  |  |
| *Netherlands (37) |  |  |  |  |
|  | Elvin Kedhi MD, PhD |  | Zwolle | Isala Klinieken (25) |
|  | Jorik Timmer, MD | Ilse Bouwhuis |  |  |
|  | Rik Hermanides, MD | Lia Nijmeijer |  |  |
|  | Eliza Kaplan, MD |  |  |  |
|  | Robert K. Riezebos, MD, PhD |  | Amsterdam | Cardio Research Hartcentrum OLVG (11) |
|  | Pouneh Samadi, MD | Jeannette, J. M. Schoep, RN |  |  |
|  | Elise van Dongen, MD | Elisabeth, M. Janzen, RN |  |  |
|  | Sander R. Niehe, MD |  |  |  |
|  | Harry Suryapranata, MD | Sandra Ahoud | Nijmegen | Radboudumc (1) |
|  | Stijn van Vugt, MD, PhD |  |  |  |
| Portugal (33) |  |  |  |  |
|  | Ruben Ramos, MD |  | Lisbon | Hospital de Santa Marta (25) |
|  | Duarte Cacela, MD |  |  |  |
|  | Ana Santana, MD |  |  |  |
|  | Antonio Fiarresga, MD |  |  |  |
|  | Lidia Sousa, MD |  |  |  |
|  | Hugo Marques, MD |  |  |  |
|  | Lino Patricio, MD | Mafalda Selas |  |  |
|  | Luis Bernanrdes, MD | Filipa Silva |  |  |
|  | Pedro Rio, MD | Cláudia Freixo |  |  |
|  | Ramiro Carvalho, MD |  |  |  |
|  | Rui Ferreira, MD |  |  |  |
|  | Tiago Silva, MD |  |  |  |
|  | Ines Rodrigues, MD |  |  |  |
|  | Pedro Modas, MD |  |  |  |
|  | Guilherme Portugal, MD |  |  |  |
|  | Jose Fragata, MD |  |  |  |
|  | Fausto J. Pinto, PhD | Inês Zimbarra Cabrita, PhD | Lisbon | Santa Maria University Hospital, Cardiology Department, CHLN (6) |
|  | Miguel Nobre Menezes, MD | Andreia Rocha, MSc |  |  |
|  | Guilhermina Cantinho Lopes, MD | Francisca Patuleia Figueiras, PhD |  |  |
|  | Ana Gomes Almeida, PhD | Andreia Coelho, BSc |  |  |
|  | Pedro Canas Silva, MD | Marta Capinha |  |  |
|  | Angelo Nobre, MD | Maria Inês Caetano |  |  |
|  | Ana Rita Francisco, MD | Susana Silva |  |  |
|  | Nuno Ferreira, MD |  | Vila Nova de Gaia | Centro Hospitalar de Vila Nova de Gaia/Espinho, EPE (2) |
|  | Ricardo L. Lopes, MD |  |  |  |
| Argentina (29) |  |  |  |  |
| Country Leader |  |  |  |  |
| Rafael Diaz, MD *(past)* |  |  |  |  |
|  | Luis Guzman, MD | Veronica Tinnirello | Cordoba | Instituto Medico DAMIC (11) |
|  | Julio César Figal, MD | Matías Nicolás Mungo | Ciudad Autonoma de Buenos Aires | Fundación Favaloro (10) |
|  | Oscar Méndiz, MD |  |  |  |
|  | Claudia Cortés, MD |  |  |  |
|  | Roberto René Favaloro, MD |  |  |  |
|  | Carlos Alvarez, MD | Marina Garcia | Bahia Blanca | Hospital Italiano Regional del Sur Bahia Blanca (3) |
|  | Javier Courtis, MD | Valeria Godoy | Cordoba | Clinica Romagosa and Clinica De La Familia (2) |
|  | Gabriela Zeballos, MD |  |  |  |
|  | Lilia Schiavi, MD | Maria Victoria Actis | Cordoba | Clinica Del Prado (2) |
|  | Mariano Rubio, MD | Graciela Scaro, MD | Cordoba | Clínica Privada Vélez Sarsfield (1) |
| *New Zealand (28) |  |  |  |  |
| Country Leader |  |  |  |  |
| Harvey Douglas White, MD |  |  |  |  |
| Country Coordinator |  |  |  |  |
| Caroline Alsweiler |  |  |  |  |
|  | Gerard Patrick Devlin, MD | Liz Low, RN | Hamilton | Waikato Hospital (22) |
|  | Raewyn Fisher, MD | Jayne Scales, RN |  |  |
|  |  | Kirsty Abercrombie, RN |  |  |
|  | Ralph Alan Huston Stewart, MCChB, MD | Leah Howell , RN | Auckland | Auckland City Hospital (6) |
|  | Harvey Douglas White, MD | Cathrine Patten, RN |  |  |
|  | Jocelyne Benatar, MD |  |  |  |
| *Macedonia (28) |  |  |  |  |
|  | Sasko Kedev, MD, PhD |  | Skopje | University Clinic of Cardiology (28) |
|  | Irena Peovska Mitevska, MD, PhD |  |  |  |
|  | Elizabeta Srbinovska Kostovska, MD, PhD |  |  |  |
|  | Hristo Pejkov, MD, PhD |  |  |  |
| *Sweden (23) |  |  |  |  |
| Country Leader |  |  |  |  |
| Claes Held, MD, PhD |  |  |  |  |
|  | Claes Held, MD, PhD |  | Uppsala | Uppsala University (18) |
|  | Kai Eggers, MD, PHhD |  |  |  |
|  | Gunnar Frostfelt, MD, PhD | Christina Björklund, RN |  |  |
|  | Nina Johnston, MD, PhD | Maria Andreasson, RN |  |  |
|  | Maciej Olsowka, MD | Marie Essermark, RN |  |  |
|  | Axel Åkerblom, MD, PhD |  |  |  |
|  | Inga Soveri, MD, PhD |  |  |  |
|  | Johannes Aspberg, MD | Liselotte Persson | Stockholm | Karolinska Institutet at Danderyd Hospital (5) |
| Israel (15) |  |  |  |  |
| Country Leaders |  |  |  |  |
| Rafael Beyar, MD, MD, DSc, MPH | |  |  |  |
| Tali Sharir, MD |  |  |  |  |
| Country Coordinator |  |  |  |  |
| Eugenia Nikolsky, MD |  |  |  |  |
|  | Tali Sharir, MD | Or Harel, MA | Tel-Aviv | Assuta Medical Centers (9) |
|  | Dan Elian, MD |  |  |  |
|  | Arthur Kerner, MD | Margalit Bentzvi | Haifa | Rambam Medical Center (6) |
|  | Samia Massalha, MD | Ludmila Helmer |  |  |
| Japan (14) |  |  |  |  |
| Country Leader |  |  |  |  |
| Shun Kohsaka, MD |  |  |  |  |
|  | Keiichi Fukuda, MD, PhD | Ikuko Ueda, PhD | Shinjuku-ku | Keio University (7) |
|  | Shun Kohsaka, MD | Jun Fujita, MD |  |  |
|  | Satoshi Yasuda, MD, PhD | Akemi Furukawa, RN | Suita-shi | National Cerebral and Cardiovascular Center |
|  |  | Kanae Hirase, RN |  | -4 |
|  |  | Toshiyuki Nagai, MD, PhD |  |  |
|  |  | Fumiyuki Otsuka, MD, PhD |  |  |
|  | Shigeyuki Nishimura, MD | Shintaro Nakano | Hidaka | Saitama Medical University (3) |
| *Belgium (7) |  |  |  |  |
| Country Leader |  |  |  |  |
| Frans Van de Werf, MD, PhD |  |  |  |  |
| Country Coordinator |  |  |  |  |
| Kaatje Goetschalckx, MD |  |  |  |  |
|  | Kaatje Goetschalckx, MD | Valerie Robesyn | Leuven | University Hospital Leuven (7) |
|  | Frans Van de Werf, PhD, MD |  |  |  |
|  | Kathleen Claes, PhD, MD |  |  |  |
| *Taiwan (7) |  |  |  |  |
| Country Leader |  |  |  |  |
| Harvey Douglas White, MD |  |  |  |  |
| Country Coordinator |  |  |  |  |
| Caroline Alsweiler |  |  |  |  |
|  | Chung-Lieh Hung, MD | Yi-Hsuan Yang | Taipei City | Mackay Memorial Hospital (7) |
|  | Chun-Ho Yun, MD |  |  |  |
|  | Charles Jia-Yin Hou, MD |  |  |  |
|  | Jen-Yuan Kuo, MD |  |  |  |
|  | Hung-I Yeh, MD, PhD |  |  |  |
|  | Ta-Chuan Hung, MD |  |  |  |
|  | Jiun-Yi Li , MD, PhD |  |  |  |
|  | Chen-Yen Chien, MD, PhD |  |  |  |
|  | Cheng-Ting Tsai, MD |  |  |  |
|  | Chun-Chieh Liu, MD |  |  |  |
|  | Fa-Chang Yu, MD |  |  |  |
|  | Yueh-Hung Lin, MD |  |  |  |
|  | Wei-Ren Lan, MD |  |  |  |
|  | Chih-Hsuan Yen, MD |  |  |  |
|  | Jui-Peng Tsai, MD |  |  |  |
|  | Kuo-Tzu Sung, MD |  |  |  |
| *South Africa (7) |  |  |  |  |
|  | Mpiko Ntsekhe, MD |  | Cape Town | Groote Schuur Hospital / University of Cape Town (7) |
|  | Shaheen Pandie, MD | Constance Philander (Nee Talliard), ND | |  |
|  | Charle A Viljoen, MD | Noloyiso Mtana, RN |  |  |
|  | Marianne De Andrade, MD |  |  |  |
| *Switzerland (7) |  |  |  |  |
| Country Leader |  |  |  |  |
| Aldo P. Maggioni, MD |  |  |  |  |
|  | Tiziano Moccetti, MD | Adriana Anesini, RN | Lugano | Cardiocentro (7) |
|  | M.Grazia Rossi, MD | Simona Maspoli, RN |  |  |
|  |  | Manuela Mombelli, RN |  |  |
| Egypt (6) |  |  |  |  |
|  | Magdy Abdelhamid, MD | Ahmed Talaat, MD | Cairo | Cairo University (6) |
|  | Ahmed Adel, MD |  |  |  |
|  | Ahmed Kamal, MsC |  |  |  |
|  | Hossam Mahrous, MD |  |  |  |
|  | Sameh El Kaffas, MD |  |  |  |
|  | Hussien El Fishawy, MD |  |  |  |
| Romania (5) |  |  |  |  |
|  | Calin Pop, MD, PhD |  | Bucharest | Emergency County Hospital Baia Mare (4) |
|  | Matei Claudia, MD, PhD |  |  |  |
|  | Bogdan A. Popescu, MD, PhD |  | Bucharest | Emergency Institute of Cardiovascular Diseases ''Prof. Dr. C. C. Iliescu'' (1) |
|  | Carmen Ginghina, MD, PhD | Monica Rosca, MD, PhD |  |  |
|  | Dan Deleanu, MD, PhD | Carmen C. Beladan, MD, PhD |  |  |
|  | Vlad A. Iliescu, MD, PhD |  |  |  |
| *Saudi Arabia (5) |  |  |  |  |
|  | Mouaz H. Al-Mallah, MD MSc | Sarah Zahrani, RN | Central Province | King AbdulAziz Cardiac Center (5) |
|  | Ahmed Aljzeeri, MD |  |  |  |
|  | Hani Najm, MD |  |  |  |
|  | Ali Alghamdi, MD |  |  |  |
| *Peru (4) |  |  |  |  |
|  | Walter Enrique Mogrovejo Ramos, MD | Marco Antonio Monsalve Davila, RN | Mirafloes | Instituto Neuro Cardiovascular De Las Americas (4) |
| Thailand (3) |  |  |  |  |
| bCountry Leader |  |  |  |  |
| Harvey Douglas White, MD |  |  |  |  |
| Country Coordinator |  |  |  |  |
| Caroline Alsweiler |  |  |  |  |
|  | Srun Kuanprasert, MD |  | Chiang Mai | Maharaj Nakorn Chiang Mai Hospital (2) |
|  | Arintaya Prommintikul, MD |  |  |  |
|  | Weerachai Nawarawong, MD | Supatchara Khwakhong, RN |  |  |
|  | Surin Woragidpoonpol, MD | Anong Chaiyasri, RN |  |  |
|  | Thitipong Tepsuwan, MD | Warangkana Mekara, RN |  |  |
|  | Noppon Taksaudom, MD | Supap Kulthawong, RN |  |  |
|  | Chataroon Rimsukcharoenchai, MD | Anong Amaritakomol, RN |  |  |
|  | Juntima Euathrongchit, MD |  |  |  |
|  | Yutthaphan Wannasopha, MD |  |  |  |
|  | Sukit Yamwong, MD | Pachara Panpunuan, RN | Bangkok | Ramathibodi Hospital (1) |
|  | Piyamitr Sritara, MD |  |  |  |
|  | Suthara Aramcharoen, MD |  |  |  |
|  | Krissada Meemuk, MD |  |  |  |
| *Malaysia (2) |  |  |  |  |
| Country Leader |  |  |  |  |
| Harvey Douglas White, MD |  |  |  |  |
| Country Coordinator |  |  |  |  |
| Caroline Alsweiler |  |  |  |  |
|  | Ahmad Khairuddin, MD | Noor Syamira Mokhtar, RN | Kuala Lumpur | Institut Jantung Negara (2) |
|  | Hafidz Abd Hadi, MD | Nor Asiah Basri, RN |  |  |
|  | Shaiful Azmi Yahaya, MD | Irni Yusnida, RN |  |  |
|  |  | Humayrah Hashim |  |  |
|  |  |  |  |  |
| ** Countries participated in Economics Quality of Life (EQoL) Questionnaires* | | |  |  |
| ***This site received one participant in transfer that was randomized at another site* | | |  |  |

# Appendix S2: ISCHEMIA Committee, CCC, Trial-Related Personnel

| **Past and Current Committee Members** |
| --- |
| **Leadership Committee** |
| Judith S. Hochman (Chair) |
| David J. Maron (Co-Chair) |
| William Boden (Co-Principal Investigator) |
| Robert Harrington (Co-Principal Investigator) |
| Gregg W. Stone (Co-Principal Investigator) |
| David Williams (Co-Principal Investigator) |
|  |
| **Executive Committee** |
| Judith S. Hochman (Chair) |
| David J. Maron (Co-Chair) |
| Karen P. Alexander |
| Sripal Bangalore |
| Jeffrey Berger |
| William Boden |
| Robert Harrington |
| Daniel Mark |
| Sean M. O'Brien |
| Harmony R. Reynolds |
| Yves Rosenberg |
| Leslee J. Shaw |
| John Spertus |
| Gregg W. Stone |
|  |
| **Steering Committee** |
| Judith S. Hochman (Chair) |
| David J. Maron (Co-Chair) |
| *Members of Executive Committee* |
| Christie Ballantyne*** |
| Daniel Berman |
| Rafael Beyar*** |
| Balram Bhargava |
| Chris Buller*** |
| Antonio (Tony) Carvalho** |
| Bernard R. Chaitman |
| Rafael Diaz*** |
| Rolf Doerr |
| Vladimir Dzavik |
| Shaun Goodman |
| Gilbert Gosselin |
| Rory Hachamovitch*** |
| Christian Hamm*** |
| Claes Held |
| Malte Helm*** |
| Kurt Huber*** |
| Lixin Jiang |
| Matyas Keltai |
| Shun Kohsaka |
| Irene Lang*** |
| Renato Lopes |
| Jose Lopez-Sendon |
| Aldo Maggioni |
| John Mancini |
| C. Noel Bairey Merz |
| James Min |
| Eric Peterson*** |
| Michael H. Picard |
| Witold Ruzyllo |
| Joseph Selvanayagam |
| Roxy Senior |
| Tali Sharir |
| Gabriel Steg |
| Hanna Szwed |
| Frans Van de Werf*** |
| William Weintraub |
| Harvey White |
| David Williams |
|  |
| **Optimal Medical Therapy Committee** |
| William Boden (Co-Chair) |
| David J. Maron (Co-Chair) |
| Christie Ballantyne |
| Sripal Bangalore |
| Karen Calfas **** |
| Bernard R. Chaitman |
| Mary Ann Champagne |
| Michael Davidson |
| Jerome Fleg |
| Peter A. McCullough |
| Jonathan Newman |
| Peter Stone |
|  |
| **Optimal Revascularization Therapy Planning Committee** |
| Gregg W. Stone (Chair) |
| **Subcommittee: CABG** |
| Philippe Menasche (Co-Chair) |
| Sripal Bangalore |
| Michael Davidson**** |
| Stephen Fremes |
| Robert Guyton |
| Michael Mack |
| Fred Mohr |
| Anupama Rao |
| Joe Sabik |
| Oz Shapira |
| David Taggart |
| James Tatoulis |
|  |
| **Subcommittee: PCI** |
| David Williams (Co-Chair) |
| Sripal Bangalore |
| Jim Blankenship |
| Sorin Brener |
| Chris Buller |
| Antonio Colombo |
| Bernard de Bruyne |
| Philippe Généreux |
| Robert Harrington |
| Dean Kereiakes |
| Thierry Lefevre |
| Jeffrey Moses |
|  |
| **Clinical Events** |
| **Endpoint Definition Panel** |
| Bernard R. Chaitman (Chair) |
| Karen P. Alexander |
| Judith S. Hochman |
| Ken Mahaffey |
| David J. Maron |
| Gregg W. Stone |
| Harvey White |
| **Clinical Event Review Committee** |
| Bernard R. Chaitman (Chair) |
| Salvador Cruz-Flores |
| Nicholas Danchin |
| Eli Feen |
| Mario J. Garcia |
| Paul Hauptman |
| Abhay A. Laddu |
| Eugene Passamani |
| Ileana L. Pina |
| Maarten Simoons |
| Hicham Skali |
| Kristian Thygesen |
| David Waters |
| **CEC Administrative Group** |
| Karen P. Alexander |
| Patricia Endsley*** |
| Gerard Esposito |
| Jeffrey Kanters |
| John Pownall |
| Dimitrios Stournaras |
|  |
| **ISCHEMIA Imaging Committee** |
| Leslee J. Shaw (Chair) |
| Daniel Berman |
| Matthias Friedrich |
| Rory Hachamovitch |
| Raymond Kwong |
| John Mancini |
| James Min |
| Dana Oliver |
| Michael H. Picard |
| Harmony R. Reynolds |
|  |
| **Biostatistics Planning Committee** |
| Frank Harrell (Chair) |
| Jeffrey Blume |
| Kerry Lee |
| Sean M. O'Brien |
|  |
| **BioRepository Committee** |
| Jeffrey Berger (Chair) |
| Claes Held |
| Iftikhar Kullo |
| Bruce McManus |
| Kristin Newby |
|  |
| **EQOL Committee** |
| Daniel Mark (Co-Chair) |
| John Spertus (Co-Chair) |
| David Cohen |
| William Weintraub |
|  |
| **Recruitment for Women & Minorities** |
| C. Noel Bairey Merz (Chair) |
| Raffaele Bugiardini |
| Jelena Celutkiene |
| Jorge Escobedo |
| Angela Hoye |
| Radmila Lyubarova |
| Deirdre Mattina |
| Jesus Peteiro |
| Harmony R. Reynolds |
| Paola Smanio |
|  |
| **Publications** |
| David J. Maron (Chair) |
| Karen P. Alexander |
| Sripal Bangalore |
| Jeffrey Berger |
| William Boden |
| Robert Harrington |
| Judith S. Hochman |
| Sean M. O'Brien |
| Harmony R. Reynolds |
| Yves Rosenberg |
| Gregg W. Stone |
| **Publication Subcommittees** |
| **Economics** |
| Daniel Mark (Chair) |
| John Spertus |
| **QOL** |
| John Spertus (Chair) |
| Daniel Mark |
| **Stress Testing** |
| Leslee J. Shaw (Chair) |
| Dan Berman |
| Bernard R. Chaitman |
| Jerome Fleg |
| Raymond Kwong |
| Michael H. Picard |
| Harmony R. Reynolds |
| Roxy Senior |
| **CCTA** |
| James Min (Chair) |
| Jonathan Leipsic |
| John Mancini |
| **Angiography/Optimal Revascularization Therapy** |
| Gregg W. Stone (Chair) |
| Ziad Ali (Co-chair) |
| Sripal Bangalore |
| David Williams |
| (Philippe Genereux, former Chair, Angiography Subcommittee)* |
| **Optimal Medical Therapy** |
| William Boden (Co-Chair) |
| David J. Maron (Co-Chair) |
| Jerome Fleg |
| Jonathan Newman |
| **Biorepository** |
| Jeffrey Berger (Chair) |
| **CEC** |
| Bernard R. Chaitman (Chair) |
| Karen P. Alexander |
| **CKD** |
| Sripal Bangalore (Chair) |
| Karen P. Alexander |
| Jerome Fleg |
| Judith S. Hochman |
| David J. Maron |
| Roy Mathew |
| Sean M. O'Brien |
| Harmony R. Reynolds |
| Mandeep Sidhu |
| **CIAO** |
| Harmony R. Reynolds (Chair) |
|  |
|  |
| **DSMB Members** |
| Lawrence Friedman (Chair) |
| Jeffrey Anderson |
| Jessica Berg *** |
| David DeMets |
| C. Michael Gibson |
| Gervasio Lamas |
| Nicole Deming |
| Jonathan Himmelfarb |
| Pamela Ouyang |
| Pamela Woodard |
|  |
| **Independent Statistical Analysis Center for DSMB Reporting** |
| Frank Harrell |
| Samuel Nwosu |
|  |
| **NHLBI Program Staff** |
| **Project Office** |
| Yves Rosenberg (Project Officer) |
| Jerome Fleg |
| Ruth Kirby |
| **Statisticians** |
| Neal Jeffries |
|  |
| **ISCHEMIA Clinical Coordinating Center (CCC)** |
| **Study Leadership** |
| Judith S. Hochman (Study Chair, Director of CCC) |
| David J. Maron (Study Co-Chair, Co-Director of CCC, US Country Leader) |
| **CCC Faculty** |
| Sripal Bangalore (Optimal Revascularization Therapy CCC Director, Regional Leader) |
| Jeffrey Berger (Director of the Biorepository, Regional Leader) |
| William Boden (US-VA Regional Leader) |
| Jonathan Newman (Optimal Medical Therapy CCC Director, Regional Leader) |
| Harmony R. Reynolds (Associate Director of CCC, CCC Imaging Lead, Regional Leader) |
| Mandeep Sidhu (US-VA Regional Co-Leader) |
| ***Program Directors*** |
| Jean E. Denaro**** |
| Stephanie Mavromichalis |
| ***Project Managers*** |
| Kevin Chan |
| Gia Cobb* |
| Aira Contreras |
| Diana Cukali* |
| Stephanie Ferket*** |
| Andre Gabriel*** |
| Antonietta Hansen* |
| Arline Roberts |
| ***Clinical Research Associates*** |
| Michelle Chang |
| Sharder Islam* |
| Graceanne Wayser* |
| Solomon Yakubov*** |
| Michelle Yee |
| ***Clinical Trial Assistants*** |
| Caroline Callison |
| Isabelle Hogan |
| Albertina Qelaj* |
| Charlotte Pirro* |
| Kerrie Van Loo |
| Brianna Wisniewski* |
| ***Grants and Finance Administration*** |
| Margaret Gilsenan (Grants Manager) |
| Bevin Lang |
| Samaa Mohamed |
| ***Publications Team*** |
| Shari Esquenazi-Karonika (Publications Manager) |
| Patenne Mathews  Anna Naumova  Jihyun Lyo* |
| ***Data Analyst*** |
| Vincent Setang* |
| Mark Xavier* |
|  |
| **Statistical and Data Coordinating Center (SDCC)** |
| Sean M. O’Brien (Principal Investigator) |
| Karen P. Alexander (Co-Principal Investigator) |
|  |
| **Economics and Quality of Life Coordinating Center (EQOL CC)** |
| *Duke Clinical Research Institute, Durham, NC* |
| Daniel B. Mark (Principal Investigator) |
| Kevin Anstrom |
| Khaula Baloch |
| Janet Blount |
| Patricia Cowper |
| Linda Davidson-Ray |
| Laura Drew |
| Tina Harding |
| J David Knight |
| Diane Minshall Liu |
| Betsy O’Neal |
| Thomas Redick |
|  |
| *Saint Luke’s Mid America Heart Institute, Kansas City, MO* |
| John Spertus (Principal Investigator) |
| Philip Jones |
| Karen Nugent |
| Grace Jingyan Wang |
|  |
| **ISCHEMIA Imaging Coordinating Center (ICC)** |
| Leslee J. Shaw (Principal Investigator) |
| Lawrence Phillips |
| Abhinav Goyal |
| Holly Hetrick |
| Dana Oliver |
| *Nuclear Core Lab* |
| Daniel Berman (Director) |
| Sean W. Hayes (Co-Director) |
| John D. Friedman |
| R. James Gerlach |
| Mark Hyun |
| Romalisa Miranda-Peats |
| Piotr Slomka |
| Louise Thomson |
| *CMR Core Lab* |
| Raymond Y. Kwong (Director) |
| Matthias Friedrich (Director)*** |
| Francois Pierre Mongeon (Co-Director) |
| Steven Michael |
| *Echo Core Lab* |
| Michael H. Picard (Director) |
| Judy Hung |
| Marielle Scherrer-Crosbie |
| Xin Zeng |
|  |
| **ECG/ETT CoreLab** |
| Bernard R. Chaitman (Director) |
| Jane Eckstein |
| Bandula Guruge |
| Mary Streif |
|  |
| **Angiographic Core Lab** |
| Ziad Ali (Director) |
| Philippe Genereux (Director)*** |
| Maria A. Alfonso |
| Maria P. Corral |
| Javier J. Garcia |
| Jennifer Horst |
| Ivana Jankovic |
| Maayan Konigstein |
| Mitchel B. Lustre* |
| Yolayfi Peralta |
| Raquel Sanchez |
|  |
| **CCTA Core Lab** |
| James Min (Director) |
| Reza Arsanjani |
| Matthew Budoff |
| Kimberly Elmore |
| Millie Gomez |
| Cameron Hague |
| Niree Hindoyan |
| Jonathan Leipsic |
| GB John Mancini |
| Rine Nakanishi |
| M. Barbara Srichai-Parsia |
| Eunice Yeoh |
| Tricia Youn |
|  |
| **Academic Research Organizations (AROs)** |
| *Associazione Nazionale Medici Cardiologi Ospedalieri (ANMCO) -Italy & Switzerland* |
| Aldo P. Maggioni (Country Leader) |
| Francesca Bianchini |
| Martina Ceseri |
| Andrea Lorimer |
| Marco Magnoni |
| Francesco Orso |
| Laura Sarti |
| Martinia Tricoli* |
| *Brazilian Clinical Research Institute (BCRI) - Brazil* |
| Antonio Carvalho (Country Leader)** |
| Renato Lopes (Country Leader) |
| Lilian Mazza Barbosa |
| Tauane Bello Duarte |
| Tamara Colaiácovo Soares |
| Julia de Aveiro Morata |
| Pedro Carvalho |
| Natalia de Carvalho Maffei |
| Flávia Egydio* |
| Anelise Kawakami* |
| Janaina Oliveira* |
| Elissa Restelli Piloto* |
| Jaqueline Pozzibon*** |
| *Canadian Heart Research Centre (CHRC) - Canada* |
| Shaun Goodman (Country Leader) |
| Diane Camara |
| Neamat Mowafy |
| Caroline Spindler |
| *China Oxford Centre for International Health Research - China* |
| Lixin Jiang (Country Leader) |
| Hao Dai |
| Fang Feng |
| Jia Li |
| Li Li* |
| Jiamin Liu |
| Qiulan Xie |
| Haibo Zhang |
| Jianxin Zhang |
| Lihua Zhang |
| Liping Zhang |
| Ning Zhang |
| Hui Zhong |
| *Estudios Clínicos Latino America (ECLA) - Argentina* |
| Rafael Diaz*** |
| Claudia Escobar |
| Maria Eugenia Martin* |
| Andrea Pascual* |
| *Foundation for Biomedical Research of La Paz University Hospital (FIBHULP) - Spain* |
| José Lopez-Sendon (Country Leader) |
| Paloma Moraga |
| Victoria Hernandez |
| Almudena Castro |
| Maria Posada* |
| Sara Fernandez |
| José Luis Narro Villanueva |
| Rafael Selgas |
| *French Alliance for Cardiovascular Trials (FACT) - France* |
| Gabriel Steg (Country Leader) |
| Helene Abergel |
| Jean Michel Juliard |
| *Green Lane Coordinating Centre Ltd. (GLCC) -Malaysia, New Zealand, Singapore, Taiwan, Thailand* |
| Harvey White (Country Leader) |
| Caroline Alsweiler |
| *KU Leuven Research & Development - Belgium** |
| Frans Van de Werf (Country Leader) |
| Kathleen Claes |
| Kaatje Goetschalckx |
| Ann Luyten |
| Valerie Robesyn |
| *South Australian Health and Medical Research Institute Ltd (SAHMRI) - Australia* |
| Joseph B. Selvanayagam (Country Leader) |
| Deirdre Murphy |
|  |
| **Contract Research Organizations (CROs) for ISCHEMIA Trial** |
| *FOCUS Clinical Research Center d.o.o. Belgrade - Serbia* |
| Nevena Garcevic |
| Jelena Stojkovic |
| *iProcess Global Research Inc. - India* |
| Asker Ahmed |
| Richa Bhatt |
| Nitika Chadha* |
| Vijay Kumar* |
| Sadath Lubna*** |
| Pushpa Naik |
| Shruti Pandey* |
| Karthik Ramasamy* |
| Mohammed Saleem |
| Pratiksha Sharma |
| Hemalata Siddaram* |
|  |
| **past members / past organizations*  ***deceased* |
